# Evaluation of the Possibility to Detect Circulating Tumor DNA From Pituitary Adenoma

**DOI:** 10.3389/fendo.2019.00615

**Published:** 2019-09-18

**Authors:** Kaspars Megnis, Raitis Peculis, Vita Rovite, Pola Laksa, Helvijs Niedra, Inga Balcere, Olivija Caune, Austra Breiksa, Jurijs Nazarovs, Janis Stukens, Ilze Konrade, Valdis Pirags, Janis Klovins

**Affiliations:** ^1^Human Genetics and Molecular Medicine, Latvian Biomedical Research and Study Centre, Riga, Latvia; ^2^Department of Internal Medicine, Riga Stradins University, Riga, Latvia; ^3^Institute of Pathology, Pauls Stradins Clinical University Hospital, Riga, Latvia; ^4^Department of Neurosurgery, Pauls Stradins Clinical University Hospital, Riga, Latvia; ^5^Department of Endocrinology, Pauls Stradins Clinical University Hospital, Riga, Latvia; ^6^Faculty of Medicine, University of Latvia, Riga, Latvia

**Keywords:** pituitary adenoma, circulating tumor DNA, competitive allele-specific TaqMan, next-generation sequencing, *GNAS*

## Abstract

**Objective:** Circulating free DNA (cfDNA) in general and circulating tumor DNA (ctDNA) in particular is becoming an increasingly used form of liquid biopsy biomarkers. In this study, we are investigating the ability to detect ctDNA from the plasma of pituitary adenoma (PA) patients.

**Design:** Tumor tissue samples were obtained from planed PA resections, before which blood plasma samples were taken. Somatic variants found in PA tissue samples were evaluated in related cfDNA, isolated from plasma samples.

**Methods:** Sanger sequencing, as well as previously obtained whole-exome sequencing data, were used to evaluate somatic variants composition in tumor tissue samples. cfDNA was isolated from the same PA patients and competitive allele-specific TaqMan PCR and amplicon-based next-generation sequencing (NGS) approach were used for targeted detection of variants found in corresponding tumor tissue samples.

**Results:** Using NGS-based analysis, we detected five out of 17 somatic variants in 40 to 60% of total reads, three variants in 0.50–5.00% of total read count, including *GNAS* c.601C>T, which was detected using ultra-deep NGS (1.78 million X) in 0.77% of amplicons reads. Nine variants were not detected. We also detected We were not able to detect variant found in PA tissue in cfDNA using cast-PCR, indicating that the portion of variant-containing ctDNA in total isolated cfDNA is too small to be detected with this method.

**Conclusions:** For the first time, we demonstrate the possibility to detect somatic variants of PA in cfDNA isolated from patients' blood plasma. Whether the source of variant detected in cfDNA is PA should be further tested.

## Introduction

Pituitary adenomas (PAs) are a group of diverse benign pituitary tumors associated with increased morbidity and mortality attributed to their hormonal hypersecretion, mass effects and therapy-related adverse sequelae (1). Information about PAs' prevalence varies between different studies and populations. For example, the reported prevalence of symptomatic PAs varies from 3.9 cases per 100,000 people in western Sweden (2) to 77.6 cases per 100,000 people in Banbury (Oxfordshire, UK) (1) or 115.6 cases per 100,000 people in Iceland (3). Several studies estimating the PAs' prevalence in general population disregarding their clinical significance (using radiologic or *post mortem* studies) have found that PAs are present in 10.4% (1) to 22.5% (4) of people.

The pathogenesis of sporadic PAs remains unclear. These adenomas have lower somatic variant rate than malignant tumors and variants in classic oncogenes and tumor-suppressor genes are relatively rare (5–7). Despite that, as mentioned by Caimari and Korbonits (5) and supported by Song et al. (8), somatic variants in specific genes, like, *GNAS, USP8*, or *PIK3CA*, can be found in ~40% of sporadic PAs, and in some cases, knowing the status of these somatic variants could be useful while choosing treatment options (5, 8).

Due to the relatively high prevalence of PAs and the inaccessibility of a tumor for tissue biopsies, it is useful to evaluate the circulating biomarkers in blood for PAs' diagnostics and prognostics. Circulating free DNA (cfDNA) in general and circulating tumor DNA (ctDNA) in particular is becoming a more and more popular class of liquid biopsy biomarkers (9). One of the biggest advantages of its uses as a biomarker is that cfDNA can be extracted from blood plasma, which makes sampling a minimally invasive procedure, compared to tissue biopsies (10).

ctDNA has proven itself as a useful marker in several cancer types. For instance, detection of estrogen receptor 1 (*ESR1*) variants in ctDNA can be used for assessing possible response to endocrine therapies or resistance to aromatase inhibitor therapy in breast cancer (11). In the case of lung cancer, variants in the epidermal growth factor receptor gene (*EGFR*) can be used to predict the epidermal growth factor receptor tyrosine kinase inhibitors (EGFR-TKIs) therapy effectivity (12). Whereas, in the case of colorectal cancer, ctDNA quantity can be used to detect tumor relapse in post-surgery surveillance months in advance compared to conventional follow-up (13).

However, so far, there have been conflicting results regarding the detection of ctDNA of benign neoplasms in blood plasma. There are cases where ctDNA from aldosterone-producing adenoma and benign colorectal adenomas reportedly have been detected in patients' plasma (14–16). However, other studies have failed to detect ctDNA in patients with colorectal adenomas or other neoplasms like thyroid nodules (17–19). Most of these studies have evaluated differences in the total cfDNA quantity or tried to detect tumor-specific variants in cfDNA using the PCR based methods, which does not always have the highest sensitivity comparing to next-generation sequencing (NGS) (20, 21). As benign neoplasms have a potentially lower fraction of ctDNA in plasma compared to cancers, methods that provide higher sensitivity might be needed for comprehensive analysis in benign tumors. Recently, it has been reported that even 0.3% variant allele fractions of ctDNA can be detected by specifically designed targeted NGS on Illumina NextSeq 500 (22).

The variants in the guanine nucleotide-binding protein, alpha stimulating complex locus (*GNAS*) gene are frequently encountered in PA tissue material studies. It is reported that about 40% of somatotropinomas and with lower frequency also other types of PAs have *GNAS* variants and these alterations affect clinical characteristics of the disease (23–25). Therefore, the ability to detect the *GNAS* variants in the blood of PA patients can serve as a tool for the detection of prognostic markers.

No studies on the analysis of cfDNA in case of PA have been published so far. This study is the first to investigate the ability to detect ctDNA from the plasma of PA patients. Our strategy includes the use of competitive allele-specific TaqMan PCR (cast-PCR) and NGS for targeted detection of *GNAS* c.601C>T variant. In addition, we employed NGS for a targeted search of other adenoma specific variants identified from whole-exome sequencing (WES) of germline—tumor DNA pairs from the corresponding patients.

## Materials and Methods

### Patients, Tissue, and Blood Samples

All samples and clinical information were obtained from patients who underwent planned PA resections at Pauls Stradins Clinical University Hospital and Riga East University Hospital. All patients were recruited to the Genome Database of the Latvian population (LGDB), a government-funded national biobank (26). Two informed consents were obtained from each patient after full explanation of the purpose and nature of all procedures used, broad consent for LGDB for use of biological material and medical data for human health and hereditary research, and project-specific consent to the research of pituitary tumors. Both biobank and PA research study have been approved by the Central Medical Ethics Committee of Latvia (protocol No. 22.03.07/A7 and 01.29.1/28, respectively). Sample obtainment and research process comply with the Declaration of Helsinki.

All patients (Table 1) had macroadenomas, eight of them had confirmed extrasellar adenomas, but two patients had endosellar adenomas. Three of them had clinical manifestations characteristic to somatotropinomas and seven had clinical manifestations characteristic to non-functioning pituitary adenomas. According to IHC(P) analysis of *PIT1, SF1*, and *TPIT* transcription profiles, HA074, HA090, and HA091 were predominantly of *PIT1* lineage, HA065, and HA067 were predominantly of *SF1* lineage, HA066 was predominantly of *TPIT* lineage but HA068, HA069, HA070, and HA073 had no data about expression of above-mentioned transcription factors. Four of them were females, and six were males. Their age distribution varied from 40 to 74 years. For eight patients this was their first-time diagnosis of pituitary adenoma, but for two patients this was relapse from previously operated PA. One patient had somatostatin and dopamine analog therapy before resection, but the rest of the patients had no medicamentous therapy before surgery.

**Table 1:** Study sample description.

**Patient**	**Age range**	**Diagnosis according to clinical features**	**Expressed transcription factor**	**Extrasellar extension**	**Primary PA or relapse**	**Medical therapy before sampling**	**sDNA Sanger sequencing results**	**cfDNA analysis method**	**Number of variants in sDNA from WES**	**Transcripts obtained for NGS analysis**
HA065	6−69	NFPA	SF1	Yes	Primary	No	No variant	NGS	272	3
HA066	50–59	NFPA	TPIT	Yes	Primary	No	No variant	NGS	671	2
HA067	70–79	NFPA	SF1	Yes	Relapse	No	No variant	NGS	6	5
HA068	60–69	NFPA	nd	Yes	Primary	No	No variant	NGS	1	0
HA069	60–69	NFPA	nd	Yes	Primary	No	No variant	NGS and cast-PCR	4	0
HA070	70–79	NFPA	nd	Yes	Relapse	No	No variant	NGS and cast-PCR	12	4
HA073	50–59	NFPA	nd	Yes	Primary	No	No variant	NGS and cast-PCR	10	2
HA074	40–49	GHPA	PIT1	No	Primary	Yes	*GNAS* c.601C>T	cast-PCR	3	–
HA090	50-59	GHPA	PIT1	No	Primary	No	*GNAS* c.601C>T	NGS & cast-PCR	2	1
HA091	40-49	GHPA	PIT1	Yes	Primary	No	*GNAS* c.601C>T	cast-PCR	5	–

*sDNA, somatic DNA; cfDNA, circulating free DNA; WES, whole-exome sequencing; IHC(P), immunohistochemical analysis done on formalin-fixed paraffin-embedded samples; GHPA, somatotropinoma; NFPA, non-functional pituitary adenoma; nd, no data, not performed*.

Whole blood samples were collected in EDTA vacutainers from patients before resection and in multiple cases 24 h after resection as well. Plasma samples were obtained by centrifuging whole blood for 10 min, at 2,000 g, in room temperature. After that, upper plasma layers were transferred to new 15 ml tubes and centrifuged for 10 min, at 4,000 g, in room temperature in order to remove remaining blood cells and prevent future cfDNA contamination with germline DNA (gDNA). Obtained plasma samples were aliquoted per 1 ml and frozen in −80°C for later use within 2 h after whole blood sampling.

After resection, adenoma tissue samples were carefully separated with a scalpel by the surgeon from any accompanying tissue lesions or particles that have originated from adenoma or neighboring tissue during surgery and adhered to the tumor sample. The sample was submerged in RNAlater® Solution (Thermo Fisher Scientific, USA) for later nucleic acid extraction.

### DNA Extraction

cfDNA was extracted from 2 ml of plasma using QIAamp Circulating Nucleic Acid Kit (Qiagen, Germany) following the manufacturer's instructions. Extraction outcome was evaluated and cfDNA concentration measured using Agilent High Sensitivity DNA kit and Bioanalyzer 2100 (Agilent Technologies, USA).

gDNA was acquired from LGDB [details on the sample preparation and DNA extraction are described by Rovite et al. (26)]. Somatic DNA (sDNA) was extracted from tissue samples using AllPrep DNA/RNA Mini Kit (Qiagen, Germany), following the manufacturer's instructions. The concentration of extracted gDNA and sDNA was measured using Qubit™ dsDNA HS Assay Kit and Qubit 2.0 Fluorometer (Thermo Fisher Scientific, USA).

### Sanger Sequencing and Cast-PCR

Sanger sequencing was applied to patients' sDNA and gDNA to estimate the presence of *GNAS* c.601C>T (NC_000020.11:g.58909365C>T) and c.680A>T (NC_000020.11:g.58909541A>T) variants. DNA was amplified using GNAS_Fw, GNAS_Rs, GNAS227_Fw and GNAS227_Rs primers (Supplementary Table 1) and sequenced using BigDye chemistry and ABI PRISM 3130xl Genetic Analyzer (Thermo Fisher Scientific, USA). All chromatograms were manually inspected using Finch TV software (Geospiza Inc., USA).

Cast-PCR was performed on sDNA and cfDNA from selected samples, using TaqMan® Mutation Detection Mutant Allele Assay GNAS_27887_mu /c.601C>T/ (Thermo Fisher Scientific, USA) and ViiA7 Real-Time PCR System (Thermo Fisher Scientific, USA). Twenty nanogram of sDNA samples were used for the reaction, as recommended by the manufacturer. Since cfDNA amount that could be extracted from blood plasma was too low to meet the manufacturers' recommendations for cast-PCR reaction input, 2 μl of cfDNA samples were used for the reaction, regardless of cfDNAs' concentration. All reactions were done in triplicates.

### cfDNA Amplification and NGS

For this study, we used data from WES study (to be published separately), analyzing DNA samples from peripheral blood and pituitary tumor obtained from 10 PA patients. DNA libraries were prepared with Illumina Nextera TruSeq Exome kit (Illumina, USA) following manufacturer's instructions and sequencing was performed with Illumina NextSeq 500/550 High Output v2 kit (150 cycles) (Illumina, USA). Paired-end sequencing with read length 75 bp was carried out on Illumina NexSeq 500 sequencer (Illumina, USA). Illumina exome target manifest TruSeq Rapid Exome TargetedRegions v1.2 (Illumina, USA) was used to define exome regions and Illumina Basespace Enrichment App (v3.0.0) was used for data analysis. Variant analysis revealed a median of 10 somatic variants per tumor. All variants selected for this study were found in sDNA, but not in gDNA, as well as they were validated with Sanger sequencing (Supplementary Figure 2). Only those somatic variants that were detected in sDNA of each individual patient were tested in their corresponding cfDNA. Insertions and deletions were not considered. Initially, we selected all validated variants with the exception for HA065 and HA066, where due to the high number of somatic variants three variants from each sample were selected. In addition, variant in GNAS and VPS13D genes that have been previously reported in relation to PA were included. This resulted in total of 24 variants. We were able to obtain good quality PCR fragments from cfDNA for 17 of these variants that were further used in the study.

Primers (Supplementary Table 1) were designed according to the variant positions found in whole-exome NGS using Primer3plus (available at http://www.bioinformatics.nl/cgi-bin/primer3plus/primer3plus.cgi). Primer sequences were checked for unique human genome binding with UCSC *In-Silico* PCR (available at http://rohsdb.cmb.usc.edu/GBshape/cgi-bin/hgPcr). Amplicons were designed not to surpass the average length of cfDNA fragment (165 bp).

Amplification of cfDNA regions, containing studied variants, was done using HOT FIREPol® (Solis BioDyne, Estonia). PCR products were visualized in 1.2% agarose gel.

Due to different amplicon sizes (105, 111, and 119 bp), they were divided into three libraries for cfDNAs' NGS, which were prepared with Ion Plus Fragment Library Kit (Thermo Fisher Scientific, USA). Size selection and clean-up between library preparation in all stages, except during end repair, was performed with NucleoMag® NGS Clean-up and Size beads (Macherey-Nagel, Germany). Since NucleoMag® beads guaranteed size selection is between 150 and 800 bp, during end repair stage NucleoMag® beads were replaced with Sephadex™ G-50 Fine (GE Healthcare, USA) in order to avoid excessive fragment loss as our fragments were between 105 and 136 bp long. After ligation of barcode and adapters, size selection was done with BluePippin Automated DNA Size Selection System (Sage Science, USA) to decrease the risk of contamination by adapter dimers. Quality and quantity of prepared libraries were verified with High Sensitivity DNA Reagents kit, High Sensitivity DNA Chips and Agilent 2100 Bioanalyzer (Agilent Technologies, USA). Preparation of template-positive ion PI™ Ion Sphere™ Particles was done on Ion OneTouch™ 2 instrument and Ion PI™ Hi-Q™ OT2 Solutions 200 kit (Thermo Fisher Scientific, USA). Prepared libraries were sequenced using Ion PI™ Hi-Q™ Sequencing 200 Solution kit, Ion PI™ Chip Kit V3 and Ion Proton System (Thermo Fisher Scientific, USA). 7 pMol of each library were used for sequencing, generating from 73,356 to 1,856,095 reads per library.

Sequences from Ion Proton system were exported in fastq format. Sequences were aligned to GRCh37—hg19 using BWA (v 0.7.17-r1188) (27). Position statistics were calculated with Samtools (v 0.1.19) (28). Alignments were reviewed with IGV (v 2.3.14) (Broad Institute, USA) (29).

## Results

### Sanger Sequencing and Cast-PCR

Both gDNA and sDNA obtained from 10 PA patients samples were analyzed for the presence of *GNAS* c.601C>T and c.680A>T somatic variants in tumor tissue by direct PCR amplification of corresponding DNA fragment and subsequent Sanger sequencing. The *GNAS* c.601C>T variant was present in three tumor samples while none of the gDNA samples contained this variant. None of the analyzed tumor tissue samples contained GNAS c.680A>T somatic variant.

After determining the presence of tumor-specific c.601C>T variant in tissue samples, we examined the ability of cast-PCR to detect this variant in the cfDNA obtained from the plasma of the corresponding patients. Amount of the cfDNA that we were able to extract from 2 ml blood plasma varied from 279.20 pg to 4.49 ng (13.96–224.40 pg/μl) (Supplementary Figure 1). None of the samples met the manufacturer's requirements for cfDNA input amount for the cast-PCR (20 ng). Therefore, we used the maximum amount (2 μl) of cfDNA per reaction, regardless of its concentration. That constitutes a 44–716 times lower amount of input DNA compared to the recommended one. sDNA from variant-positive tissue samples were used as positive control while sDNA from variant-negative tissue samples and related cfDNAs were used as a negative control. As a result, we were able to obtain a positive signal only from positive controls (Ct = 28.21 ± 0.90). No signal was obtained from neither negative controls nor cfDNA from *GNAS* c.601C>T positive samples, indicating that the portion of *GNAS* c.601C>T variant-containing ctDNA in total isolated cfDNA is too small to be detected with cast-PCR or cfDNA does not contain ctDNA of PA origin.

### NGS Based Analysis

For this study, we used WES data to identify cfDNA amplicon targets. A number of identified somatic variants per patient, in their sDNA but not gDNA, as well as the number of tumor variants selected for this study, are shown in Table 1. Of the 24 somatic variants, 17 amplicons encompassing their location were obtained from cfDNA while amplification of seven cfDNA regions with somatic variants was not successful. All 17 obtained amplicons were successfully sequenced with NGS. 82.35% of NGS reads mapped to the target region with an average mapping quality 57.0. The obtained depth of coverage at the variant sites ranged from 444 X to 13,048 X (median 4,833 X).

In a result, eight out of 17 analyzed variants were convincingly detected in cfDNA amplicons. Results of the cfDNA sequencing can be divided into three categories with respect to the variant frequency in the reads (Table 2). The first group includes five variants in genes *VPS13D, LDLRAD2, SPEN, GPATCH4*, and *G6PC2* that were detected between 40 and 60% of the total reads obtained from corresponding amplicons. Interestingly, all these five variants were identified in two individuals that also harbored the highest number of total somatic variants (272 and 671) in tumor tissue as identified by WES. The second group includes three variants from three individuals in genes—*MPRIP* and *RYR* (frequencies of 3.65 and 2.27%, respectively) who were identified in sDNA, but not in gDNA in previously mentioned WES study, and *GNAS* (0.77% frequency) who were identified in sDNA, but not in gDNA in Sanger sequencing, mentioned in section Sanger sequencing and cast-PCR (Supplementary Figure 2). The third group includes identified somatic variants in genes *CLEC1B, ATF4, CLCNKA, SMARCAD1, PDE3A, MTFMT, CCDC138, FXR1*, and *PRPF8* that were either not detected in any of the NGS reads or detected in a small fraction of reads, ranging from 0.02to 0.15%. It should be noted that the substitution of the different nucleotides can be found across all positions examined or any other position of the alignment that corresponds to the rate of the technical error of the used sequencing platform which at this study were 0.22%.

**Table 2:** Summary of NGS results.

**Patient ID**	**Gene**	**Position of variant**	**Wt/alt allele**	**Total coverage**	**Coverage**			
					**A**	**C**	**G**	**T**
**Alternative allele content between 40.00 and 60.00% of the total reads**								
HA065	VPS13D	chr1:12368563	C/T	10,585	6	*5,420*	11	**5,148 (48.63%)**
	LDLRAD2	chr1:22150575	C/T	7,641	3	*4,033*	8	**3,597 (47.07%)**
	SPEN	chr1:16256795	A/G	444	*225*	0	**218 (49.10%)**	1
HA066	GPATCH4	chr1:156565233	C/T	8,726	9	*3,611*	1	**5,105 (58.50%)**
	G6PC2	chr2:169764546	C/G	1,567	0	*869*	**697 (44.48%)**	1
**Alternative allele content between 0.50 and 5.00% of the total reads**								
HA073	MPRIP	chr17:17068722	C/T	3,066	0	*2,949*	5	**112 (3.65%)**
HA070	RYR1	chr19:38964226	G/A	882	**20 (2.27%)**	0	*862*	0
HA090	GNAS	chr 20:58909365	C/T	1,744,103	76	*1,730,527*	118	**13,382 (0.77%)**
**No alternative allele or at the same frequency as technological errors**								
HA067	CLCNKA	chr1:16358671	C/G	1,666	0	*1,664*	**0**	2
	SMARCAD1	chr4:95174031	A/G	8,968	*8,958*	2	**8**	0
	PDE3A	chr12:20774260	G/A	13,014	**5**	1	*13,008*	0
	MTFMT	chr15:65316064	A/T	5,964	*5,950*	0	13	**1**
	PRPF8	chr17:1563731	A/G	8,239	*8,227*	0	**12**	0
HA070	CCDC138	chr2:109473249	G/A	4,833	**4**	0	*4,829*	0
	FXR1	chr3:180633413	T/A	3,117	**1**	6	1	*3,109*
	CLEC1B	chr12:10149329	G/A	2,560	**0**	0	*2,559*	1
HA073	ATF4	chr22:39918305	C/A	1,336	**0**	0	*1,336*	0

*wt, wild-type allele; alt, alternative allele. Bold indicates coverage of alternative alleles, italic indicates coverage of wild-type alleles*.

## Discussion

During recent years, liquid biopsies and cfDNA has been studied in relation to different tumors and other diseases (30, 31). Despite the theoretically high prevalence of PAs and the broad effect on the organism caused by these tumors (32, 33), there are no studies that would examine the usability of ctDNA in PA diagnostics and prognostics. In our knowledge, this is the first study that attempts to detect ctDNA of PA origin in the patient's bloodstream.

cfDNA has been mostly studied as a prognostic tumor marker in malignant tumor types. It is widely reported that cfDNA amount in patients' bloodstream is significantly higher in cancer patients than a healthy subject, with higher amounts indicated in latter tumor stages compared to early (34–36). In other studies, researchers have applied WES to cfDNA and matching tumor samples, and they had found that cfDNA represents the genomic characteristics of a tumor (37, 38). In benign tumors, ctDNA detection has proven to be more challenging as in some cases this has been successful (14–16), but in others not (17–19).

Apoptosis, necrosis and possibly active secretion have been thought to be the main mechanisms of cfDNA release in the blood (39). However, a recent study has highlighted a stronger correlation of ctDNA detection with cellular proliferation than with cell death (40). It can be further theoreticized, that in the case of fast-growing cancers, rapid cell proliferation could result in relatively higher amounts of ctDNA released in the blood, which can be further isolated and analyzed. However, since the benign tumors generally have slower growth and consequently the proliferation rate, ctDNA could be released in lower amounts, resulting in a lower concentration in blood. For this reason, in case of the benign tumors, ctDNA could be much harder to detect and analyse. On the other hand, many pituitary cells being secretory by nature may contribute to the appearance of cfDNA from these cells into plasma. So far, several attempts have been published to analyse variants in ctDNA from benign tumors of different origin. Lupo et al. measured ctDNA yield in blood plasma from patients with thyroid nodules and analyzed variant content, using commercial assay and NGS. Although they managed to detect ctDNA in two patients, authors concluded that neither cfDNA measurements nor ctDNA variants detection were sensitive or specific enough to be used in clinical practice instead of analysis of tissue biopsy (19).

In our first attempt to analyse ctDNA from PAs, we have chosen well-known recurring somatic variant (c.601C>T) in the *GNAS* gene and succeeded to identify three tumors from PA patients with this variant. We used cast-PCR and allele-specific TaqMan assay to detect this variant in the cfDNA obtained from the plasma of the corresponding patients. Although cast-PCR is reported to have 0.10–1.00% variant detection limit in cfDNA (21), we were not able to find the presence of c.601C>T variant in any of these samples. Cast-PCR sensitivity can be affected by the fact, that amount of cfDNA input that we used was lower than manufacturer's recommended, due to a limited amount of cfDNA that we were able to extract from blood plasma. It is, of course, plausible that the amount of ctDNA that comes from PA in the bloodstream could be too low to be detected with this method because PAs are generally slow-growing benign tumors.

The method described above, however, has significant limitations. First, only patients with specific *GNAS* variant are investigated. Secondly, also the number of genomic sites per tumor/patient is limited to one. We, therefore, used NGS based sequencing of amplicons from WES determined genomic regions to estimate and quantify the possible presence of ctDNA in eight patients. This approach requires the prior knowledge of patient specific somatic variants found in tumor tissue. In our case, we selected variants identified in WES and Sanger sequencing study analyzing gDNA obtained from peripheral blood cells and sDNA from a pituitary tumor obtained from the same PA patients. In total 17 somatic variants, that were found in sDNA, but not in gDNA, were analyzed in six patients from corresponding cfDNA amplicons. From all analyzed variants the GNAS gene variant has been reported in several other studies, as mentioned above (7, 23–25). Notably, two genes VPS13D and RYR1 have also been reported to contain somatic variants in GH secreting PA tissue (41), but the localization of the variants was different than we found in our study. Further investigation is needed to explore the potential functional role of these genes in PA development.

In three patients majority of somatic variants were not detectable at the convincing level and only three variants were detected between 0.5 and 5.0% of the total reads that in general corresponds to the levels of a tumor-specific DNA found in the cfDNA shown in the studies exploring different cancers. One of these variants was c.601C>T that could not be identified with cast-PCR and allele-specific TaqMan assay. From these results, we conclude that the concentration of ctDNA in case of PAs are at the boundary of detection with semiconductor sequencing technologies and below the boundary for detection with cast-PCR and TaqMan technologies. Although the results of this study indicate successful detection of the variants in plasma-derived cfDNA in some cases, the read count obtained from the sequencing is on the borderline of sensitivity and cannot be used for tumor variant monitoring in a clinical setting with the presented technology. Potentially, other molecular techniques, for example, digital droplet PCR could be further studied to develop more sensitive monitoring methods.

The results obtained from two patients included in the study are unexpected. First of all, tumors from these patients are characterized with a higher number of somatic variants (272 and 671) than usually observed in benign PAs, but no clinical manifestations indicating increased aggressiveness or recurrent pathological tumors were identified in these patients. All five variants from these patients that we were able to detect had between 40 and 60% of the total reads that is unusually high for a ctDNA. One can hypothesize that high amount of genetic changes in these tumors could contribute to mechanisms that are related with ctDNA release into the bloodstream or these variants could be present in other tissues in patients bodies with high activity of mechanisms that promote cfDNA release.

In summary, for the first time, we demonstrate the possibility to detect somatic variants of PA in cfDNA isolated from patients' blood plasma. Beside others, we were able to detect variant in the *GNAS* gene, whose impact on PA has been widely described in the literature. Whether the source of variant detected in cfDNA is PA should be further tested.

## Data Availability

Circulating free DNA amplicon sequencing data can be found in public repository under the following links: http://www.ncbi.nlm.nih.gov/bioproject/546469; https://www.ncbi.nlm.nih.gov/biosample/11959605; https://www.ncbi.nlm.nih.gov/biosample/11959606; https://www.ncbi.nlm.nih.gov/biosample/11959607.

## Ethics Statement

The studies involving human participants were reviewed and approved by Central Medical Ethics Committee of Latvia (protocol No. 22.03.07/A7 and 01.29.1/28). The patients/participants provided their written informed consent to participate in this study.

## Author Contributions

KM participated in study design and organization of experiments and performed manuscript writing. RP participated in study design and data analysis. VR, IK, and VP participated in study design and manuscript editing. PL performed DNA isolation, Sanger sequencing, and cast-PCR. HN performed DNA isolation, amplification and next-generation sequencing. IB performed patient enrolment in the study. OC performed clinical data obtainment and description. AB and JN performed tumor tissue immunohistochemical characterization. JS performed tumor resection and tumor material obtainment. JK was the project leader, performed study design, and manuscript editing.

### Conflict of Interest Statement

The authors declare that the research was conducted in the absence of any commercial or financial relationships that could be construed as a potential conflict of interest.

## Supplementary Material

Supplementary Figure 1 Size and concentration of two representative cfDNA samples that have been extracted from 2 ml of PA patients' blood plasma and eluted in 20 μl. Measurement was done using Agilent High Sensitivity DNA kit and Bioanalyzer 2100 (Agilent Technologies, USA). (1) indicates cfDNA sample, (2) indicates High Sensitivity DNA ladder lower marker, (4) indicates High Sensitivity DNA ladder upper marker. In both samples, cfDNA size was around 170 bp, but concentration warried from 13.96 to 224.40 pg/μl.

Supplementary Figure 2 Validation of LDLRAD2, SPEN, VPS13D, G6PC2, GPATCH4, CLCNKA, MTFMT, PDE3A, PRPF8, SMARCAD1, CCDC138, CLEC1B, FXR1, RYR1, ATF4, MPRIP gene variants with Sanger sequencing in pituitary adenoma somatic (PA) DNA and patients germline DNA from samples HA065, HA066, HA067, HA070, HA073, and HA090.

Supplementary Table 1 Used primers (Metabion international AG, Germany).

## References

[1] Fernandez A Karavitaki N Wass JAH. Prevalence of pituitary adenomas: a community-based, cross-sectional study in Banbury (Oxfordshire, UK). Clin Endocrinol. (2010) 72:377–82. 10.1111/j.1365-2265.2009.03667.x 19650784

[2] Tjörnstrand A Gunnarsson K Evert M Holmberg E Ragnarsson O Rosén T. The incidence rate of pituitary adenomas in western Sweden for the period 2001-2011. Eur J Endocrinol. (2014) 171:519–26. 10.1530/EJE-14-0144 25084775

[3] Agustsson TT Baldvinsdottir T Jonasson JG Olafsdottir E Steinthorsdottir V Sigurdsson G. The epidemiology of pituitary adenomas in Iceland, 1955-2012: a nationwide population-based study. Eur J Endocrinol. (2015) 173:655–64. 10.1530/EJE-15-0189 26423473

[4] Ezzat S Asa SL Couldwell WT Barr CE Dodge WE Vance ML. The prevalence of pituitary adenomas. Cancer. (2004) 101:613–9. 10.1002/cncr.20412 15274075

[5] Caimari F Korbonits M. Novel genetic causes of pituitary adenomas. Clin Cancer Res. (2016) 22:5030–42. 10.1158/1078-0432.CCR-16-0452 27742789

[6] Newey PJ Nesbit MA Rimmer AJ Head RA Gorvin CM Attar M. Whole-exome sequencing studies of nonfunctioning pituitary adenomas. J Clin Endocrinol Metab. (2013) 98:E796–800. 10.1210/jc.2012-4028 23450047 PMC4447855

[7] Ronchi CL Peverelli E Herterich S Weigand I Mantovani G Schwarzmayr T. Landscape of somatic mutations in sporadic GH-secreting pituitary adenomas. Eur J Endocrinol. (2016) 174:363–72. 10.1530/EJE-15-1064 26701869

[8] Song Z-J Reitman ZJ Ma Z-Y Chen J-H Zhang Q-L Shou X-F. The genome-wide mutational landscape of pituitary adenomas. Cell Res. (2016) 26:1255–9. 10.1038/cr.2016.114 27670697 PMC5099864

[9] Leung F Kulasingam V Diamandis EP Hoon DSB Kinzler K Pantel K Circulating tumor DNA as a cancer biomarker: fact or fiction? Clin Chem. (2016) 62:1054–60. 10.1373/clinchem.2016.260331 27259816 PMC5326709

[10] Allin DM Shaikh R Carter P Thway K Sharabiani MTA Gonzales-de-Castro D. Circulating tumour DNA is a potential biomarker for disease progression and response to targeted therapy in advanced thyroid cancer. Eur J Cancer. (2018) 103:165–75. 10.1016/j.ejca.2018.08.013 30253333

[11] Takeshita T Yamamoto Y Yamamoto-Ibusuki M Tomiguchi M Sueta A Murakami K. Analysis of ESR1 and PIK3CA mutations in plasma cell-free DNA from ER-positive breast cancer patients. Oncotarget. (2017) 8:52142–55. 10.18632/oncotarget.18479 28881720 PMC5581019

[12] Hirsch FR Varella-Garcia M Bunn PA Franklin WA Dziadziuszko R Thatcher N. Molecular predictors of outcome with gefitinib in a phase III placebo-controlled study in advanced non-small-cell lung cancer. J Clin Oncol. (2006) 24:5034–42. 10.1200/JCO.2006.06.3958 17075123

[13] Reinert T Schøler L V Thomsen R Tobiasen H Vang S Nordentoft I. Analysis of circulating tumour DNA to monitor disease burden following colorectal cancer surgery. Gut. (2016) 65:625–34. 10.1136/gutjnl-2014-308859 25654990

[14] Cangiano D Ceolotto G Caroccia B Citton M Seccia TH Rossi GP Detection of free-circulating dna in patients with aldosterone producing adenoma. J Hypertens. (2015) 33:e111–2. 10.1097/01.hjh.0000467652.92919.31

[15] Mead R Duku M Bhandari P Cree IA. Circulating tumour markers can define patients with normal colons, benign polyps, and cancers. Br J Cancer. (2011) 105:239–45. 10.1038/bjc.2011.230 21712823 PMC3142810

[16] Kopreski MS Benko FA Borys DJ Khan A McGarrity TJ Gocke CD. Somatic mutation screening: identification of individuals harboring K-ras mutations with the use of plasma DNA. J Natl Cancer Inst. (2000) 92:918–23. 10.1093/jnci/92.11.918 10841827

[17] Perrone F Lampis A Bertan C Verderio P Ciniselli CM Pizzamiglio S. Circulating free DNA in a screening program for early colorectal cancer detection. Tumori. (2014) 100:115–21. 10.1177/030089161410000201 24852853

[18] Galanopoulos M Papanikolaou IS Zografos E Viazis N Papatheodoridis G Karamanolis D. Comparative study of mutations in single nucleotide polymorphism loci of KRAS and BRAF genes in patients who underwent screening colonoscopy, with and without premalignant intestinal polyps. Anticancer Res. (2017) 37:651–7. 10.21873/anticanres.11360 28179313

[19] Lupo M Guttler R Geck Z Tonozzi TR Kammesheidt A Braunstein GD. Is measurement of circulating tumor dna of diagnostic use in patients with thyroid nodules? Endocr Pract. (2018) 24:453–9. 10.4158/EP-2017-0213 29498908

[20] Garcia J Forestier J Dusserre E Wozny A-S Geiguer F Merle P. Cross-platform comparison for the detection of RAS mutations in cfDNA (ddPCR Biorad detection assay, BEAMing assay, and NGS strategy). Oncotarget. (2018) 9:21122–31. 10.18632/oncotarget.24950 29765524 PMC5940402

[21] Han X Wang J Sun Y. Circulating tumor DNA as biomarkers for cancer detection. Genomics Proteomics Bioinforma. (2017) 15:59–72. 10.1016/j.gpb.2016.12.004 28392479 PMC5414889

[22] Plagnol V Woodhouse S Howarth K Lensing S Smith M Epstein M. Analytical validation of a next generation sequencing liquid biopsy assay for high sensitivity broad molecular profiling. PLoS ONE. (2018) 13:e0193802. 10.1371/journal.pone.0193802 29543828 PMC5854321

[23] Landis CA Harsh G Lyons J Davis RL McCormick F Bourne HR. Clinical characteristics of acromegalic patients whose pituitary tumors contain mutant Gs protein. J Clin Endocrinol Metab. (1990) 71:1416–20. 10.1210/jcem-71-6-1416 2121775

[24] Hayward BE Barlier A Korbonits M Grossman AB Jacquet P Enjalbert A. Imprinting of the G(s)alpha gene GNAS1 in the pathogenesis of acromegaly. J Clin Invest. (2001) 107:R31–6. 10.1172/JCI11887 11254676 PMC208949

[25] Efstathiadou ZA Bargiota A Chrisoulidou A Kanakis G Papanastasiou L Theodoropoulou A. Impact of gsp mutations in somatotroph pituitary adenomas on growth hormone response to somatostatin analogs: a meta-analysis. Pituitary. (2015) 18:861–7. 10.1007/s11102-015-0662-5 26115707

[26] Rovite V Wolff-Sagi Y Zaharenko L Nikitina-Zake L Grens E Klovins J. Genome Database of the Latvian Population (LGDB): design, Goals, and Primary Results. J Epidemiol. (2018) 28:353–60. 10.2188/jea.JE20170079 29576601 PMC6048300

[27] Li H Durbin R. Fast and accurate short read alignment with Burrows-Wheeler transform. Bioinformatics. (2009) 25:1754–60. 10.1093/bioinformatics/btp324 19451168 PMC2705234

[28] Li H. A statistical framework for SNP calling, mutation discovery, association mapping and population genetical parameter estimation from sequencing data. Bioinformatics. (2011) 27:2987–93. 10.1093/bioinformatics/btr509 21903627 PMC3198575

[29] Robinson JT Thorvaldsdóttir H Winckler W Guttman M Lander ES Getz G. Integrative genomics viewer. Nat Biotechnol. (2011) 29:24–6. 10.1038/nbt.1754 21221095 PMC3346182

[30] Khatami F Tavangar SM. Circulating tumor DNA (ctDNA) in the era of personalized cancer therapy. J Diabetes Metab Disord. (2018) 17:19–30. 10.1007/s40200-018-0334-x 30288382 PMC6154523

[31] Zhang K Lin G Han Y Xie J Li J. Circulating unmethylated insulin DNA as a potential non-invasive biomarker of beta cell death in type 1 Diabetes: a review and future prospect. Clin Epigenetics. (2017) 9:44. 10.1186/s13148-017-0343-5 28450972 PMC5405546

[32] Torre LA Bray F Siegel RL Ferlay J Lortet-Tieulent J Jemal A. Global cancer statistics, 2012. CA Cancer J Clin. (2015) 65:87–108. 10.3322/caac.21262 25651787

[33] Buurman H Saeger W. Subclinical adenomas in postmortem pituitaries: classification and correlations to clinical data. Eur J Endocrinol. (2006) 154:753–8. 10.1530/eje.1.02107 16645024

[34] Sozzi G Conte D Leon M Ciricione R Roz L Ratcliffe C. Quantification of free circulating DNA as a diagnostic marker in lung cancer. J Clin Oncol. (2003) 21:3902–8. 10.1200/JCO.2003.02.006 14507943

[35] Kim K Shin DG Park MK Baik SH Kim TH Kim S. Circulating cell-free DNA as a promising biomarker in patients with gastric cancer: diagnostic validity and significant reduction of cfDNA after surgical resection. Ann Surg Treat Res. (2014) 86:136–42. 10.4174/astr.2014.86.3.136 24761422 PMC3994618

[36] Shao X He Y Ji M Chen X Qi J Shi W. Quantitative analysis of cell-free DNA in ovarian cancer. Oncol Lett. (2015) 10:3478–82. 10.3892/ol.2015.3771 26788153 PMC4665352

[37] Manier S Park J Capelletti M Bustoros M Freeman SS Ha G. Whole-exome sequencing of cell-free DNA and circulating tumor cells in multiple myeloma. Nat Commun. (2018) 9:1691. 10.1038/s41467-018-04001-5 29703982 PMC5923255

[38] Song J Yang Z. Case report: whole exome sequencing of circulating cell-free tumor DNA in a follicular thyroid carcinoma patient with lung and bone metastases. J Circ biomarkers. (2018) 7:1849454418763725. 10.1177/1849454418763725 29623111 PMC5881966

[39] Wan JCM Massie C Garcia-Corbacho J Mouliere F Brenton JD Caldas C. Liquid biopsies come of age: towards implementation of circulating tumour DNA. Nat Rev Cancer. (2017) 17:223–38. 10.1038/nrc.2017.7 28233803

[40] Abbosh C Birkbak NJ Wilson GA Jamal-Hanjani M Constantin T Salari R. Phylogenetic ctDNA analysis depicts early-stage lung cancer evolution. Nature. (2017) 545:446–51. 10.1038/nature22364 28445469 PMC5812436

[41] Välimäki N Demir H Pitkänen E Kaasinen E Karppinen A Kivipelto L. Whole-genome sequencing of Growth Hormone (GH)-secreting pituitary adenomas. J Clin Endocrinol Metab. (2015) 100:3918–27. 10.1210/jc.2015-3129 26280510

