# Supplementary figures and images for: Evaluation of the Possibility to Detect Circulating Tumor DNA From Pituitary Adenoma
Source: Front Endocrinol (Lausanne). 2019 Sep 18;10:615. doi: 10.3389/fendo.2019.00615 (PMC6759656; doi:10.3389/fendo.2019.00615)

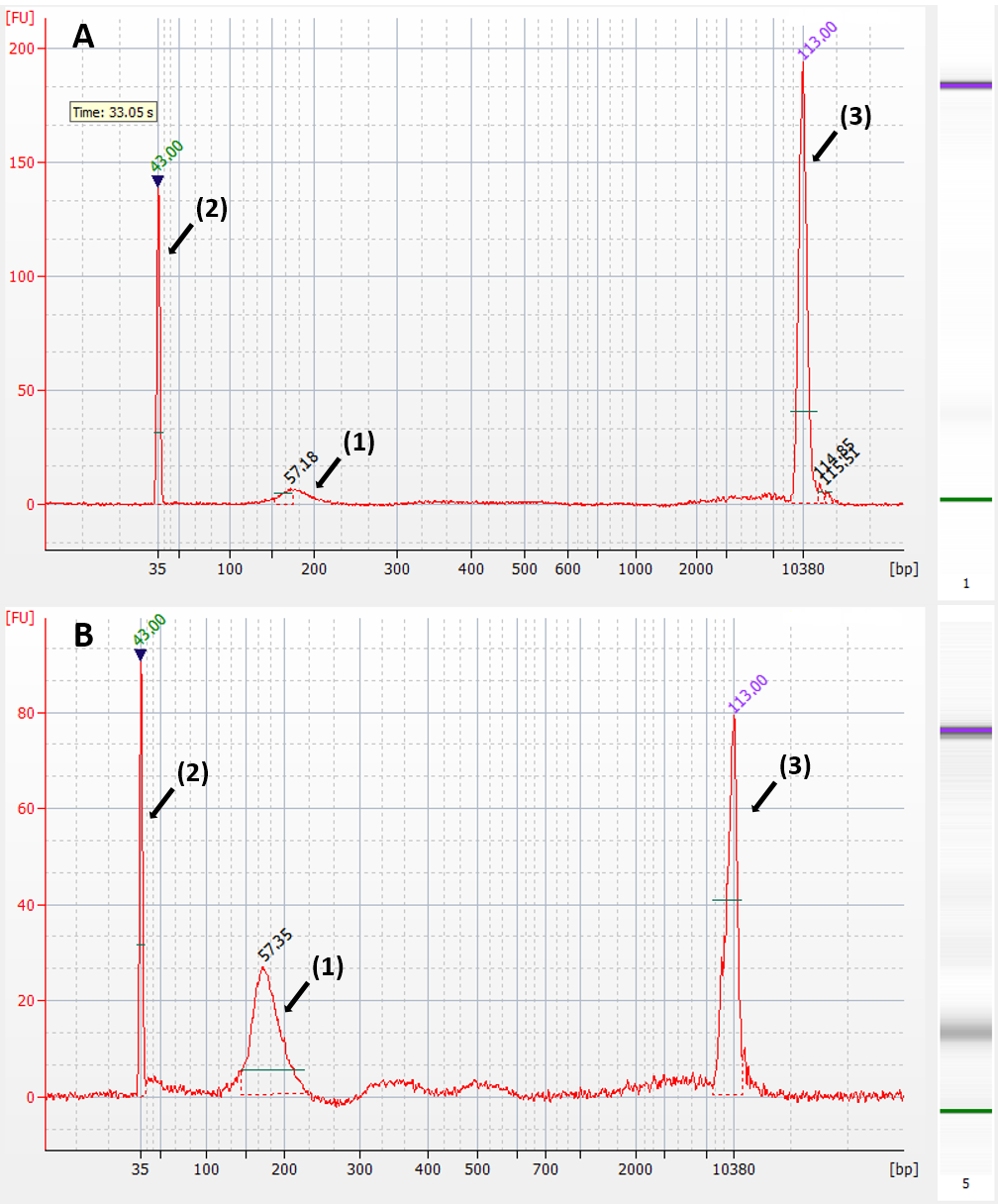

Supplement: Supplementary Figure 1 — Size and concentration of two representative cfDNA samples that have been extracted from 2 ml of PA patients' blood plasma and eluted in 20 μl. Measurement was done using Agilent High Sensitivity DNA kit and Bioanalyzer 2100 (Agilent Technologies, USA). (1) indicates cfDNA sample, (2) indicates High Sensitivity DNA ladder lower marker, (4) indicates High Sensitivity DNA ladder upper marker. In both samples, cfDNA size was around 170 bp, but concentration warried from 13.96 to 224.40 pg/μl. [file Image_1.PNG]
