# Supplementary material for: Evaluation of the Possibility to Detect Circulating Tumor DNA From Pituitary Adenoma
Source: Front Endocrinol (Lausanne). 2019 Sep 18;10:615. doi: 10.3389/fendo.2019.00615 (PMC6759656; doi:10.3389/fendo.2019.00615)

HA065 VPS13D chr1:12368563

Amplicon sequence:

>chr1:12368506-12368641 136bp

TGGATTGCGTTGTCGTGGATctccaggacatggacatctttgctgcag
agagacatccgagagaatactcgaaggcaccagaggatagtagtggagatc
tgatcttcccttcctatTTTGTGCGACAGACAGGAGG

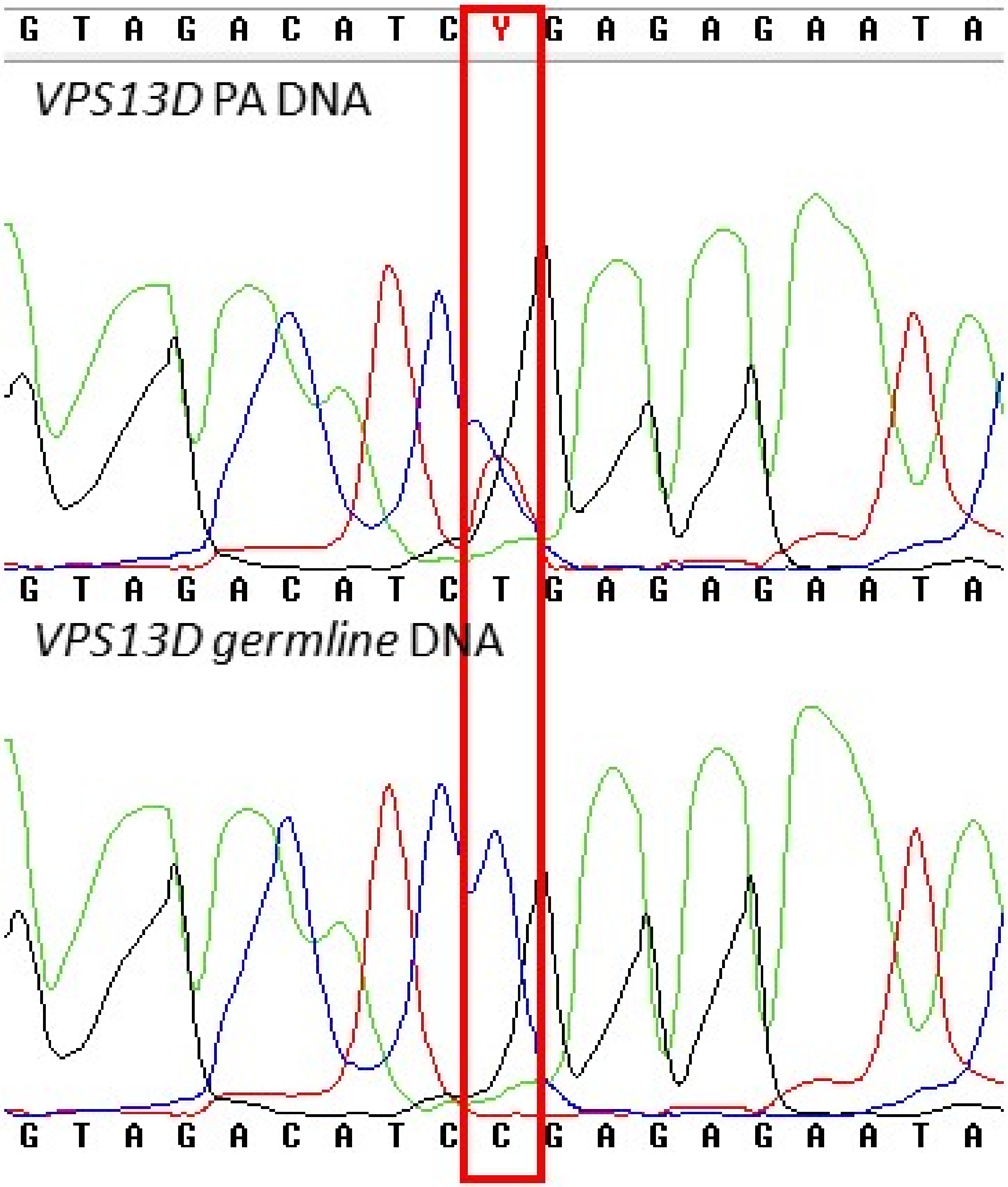

HA065 LDLRAD2 chr1:22150575

Amplicon sequence:

>chr1:22150492-22150626 135bp

CCCCTGGCTTCAAGTTCTGTctccacagagctcaatacctgcctctct
gcccatggtagggggcgtcctgccccactccagaacgctgggccccatcccg
agtgcccggcagggtcCCTTACCGCAGTGCTGTCA

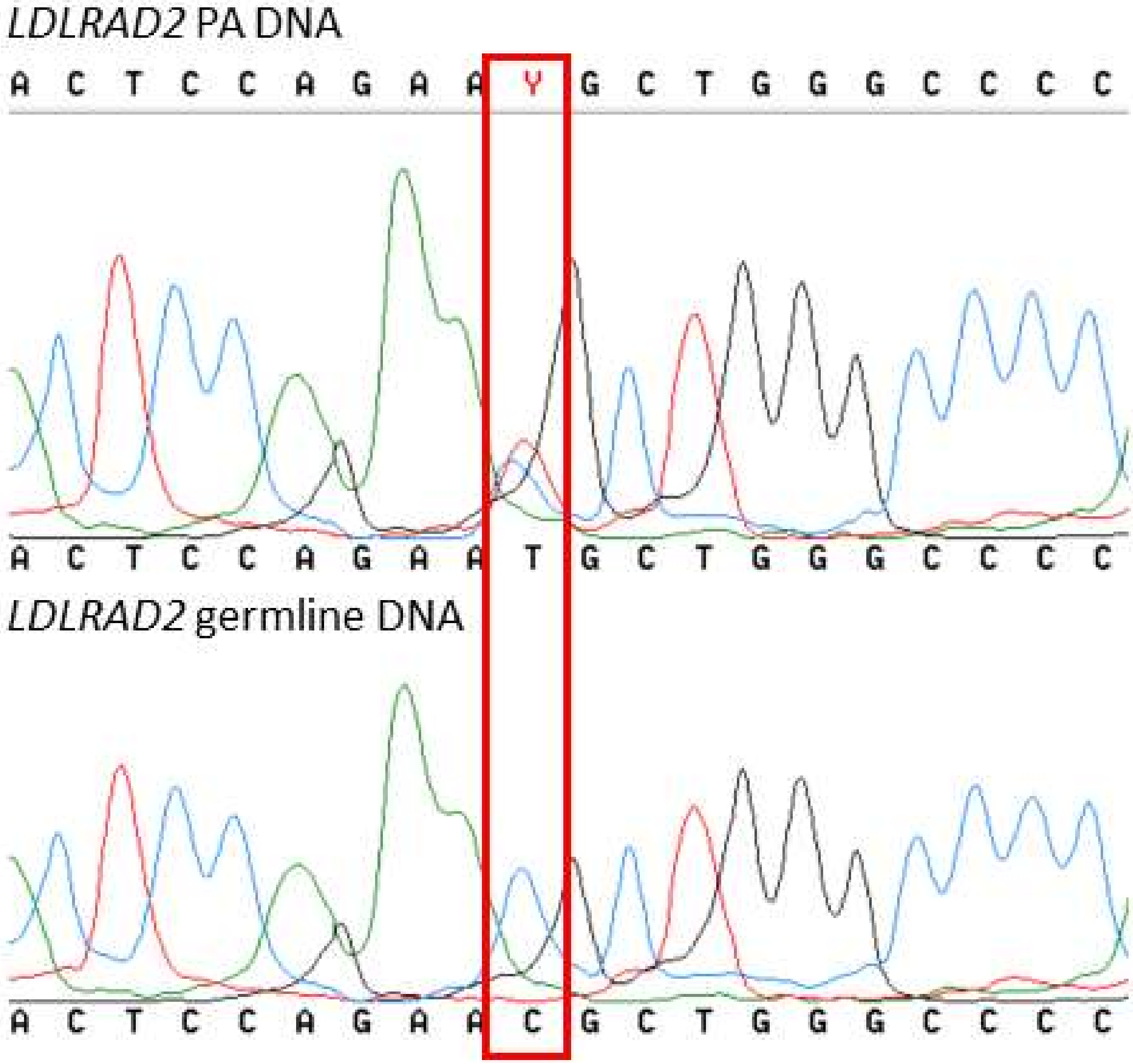

HA065 SPEN chr1:16256795

Amplicon sequence:

>chr1:16256749-16256856 108bp

TCGTTGGGACTCTCAGATGAaacaggatgctggcagatttgatgtga
gtttcccaaacagcataattaagagagatagccttcgaaaAAGGTCTGT
ACGAGATCTGGA

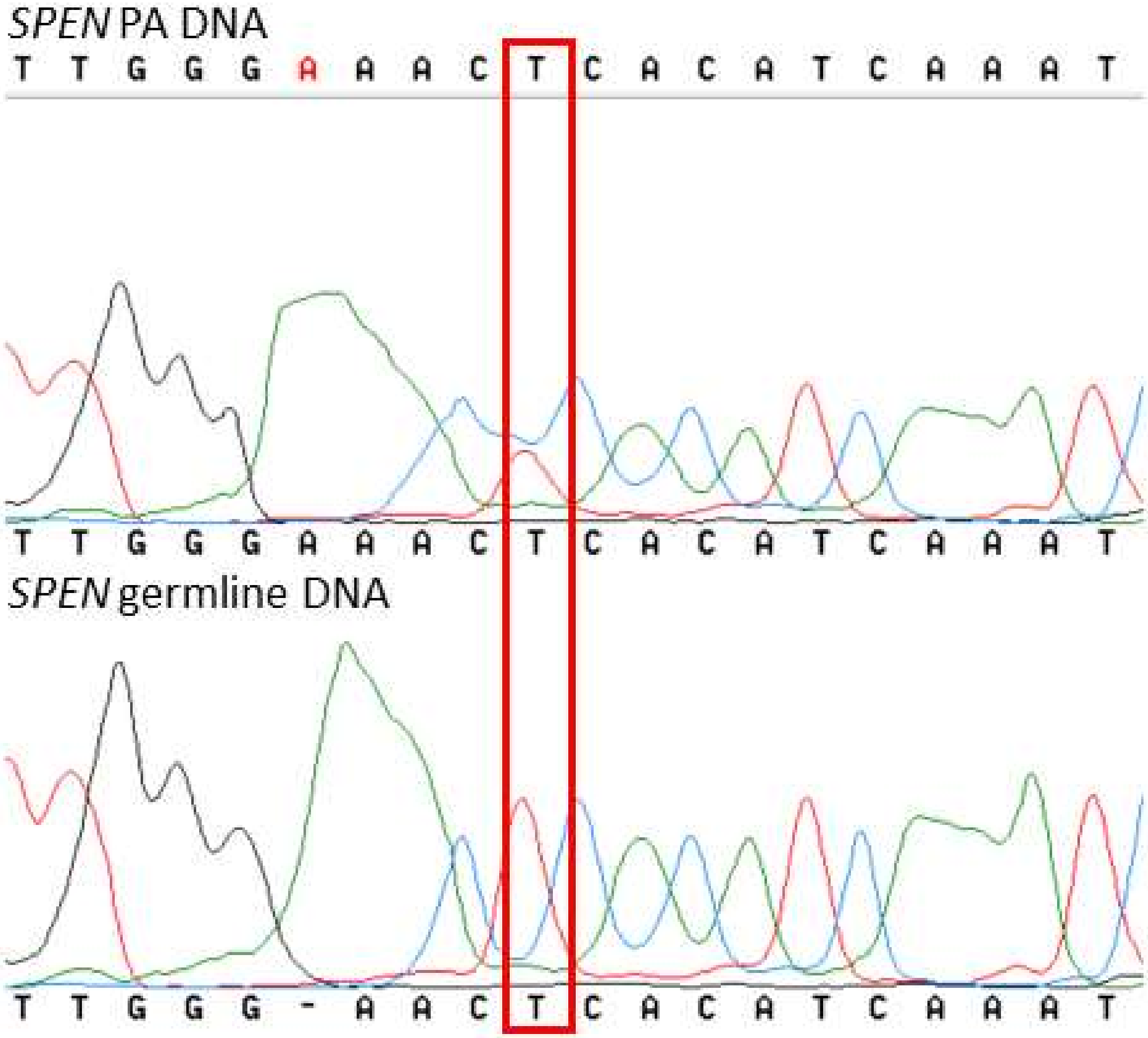

HA066 GPATCH4 chr1:156565233

Amplicon sequence:

>chr1:156565195-156565334 140bp

GGGAATGAGAAGGAGGACGCtgcaggaacaagtgggcttgggga
attgaatagcagagagcaaaccaatcagtccctcaggaaagggaagaaaa
agaagaggtggcaccatgaagaggagAAGATGGGGGTCTTGGAG
GA

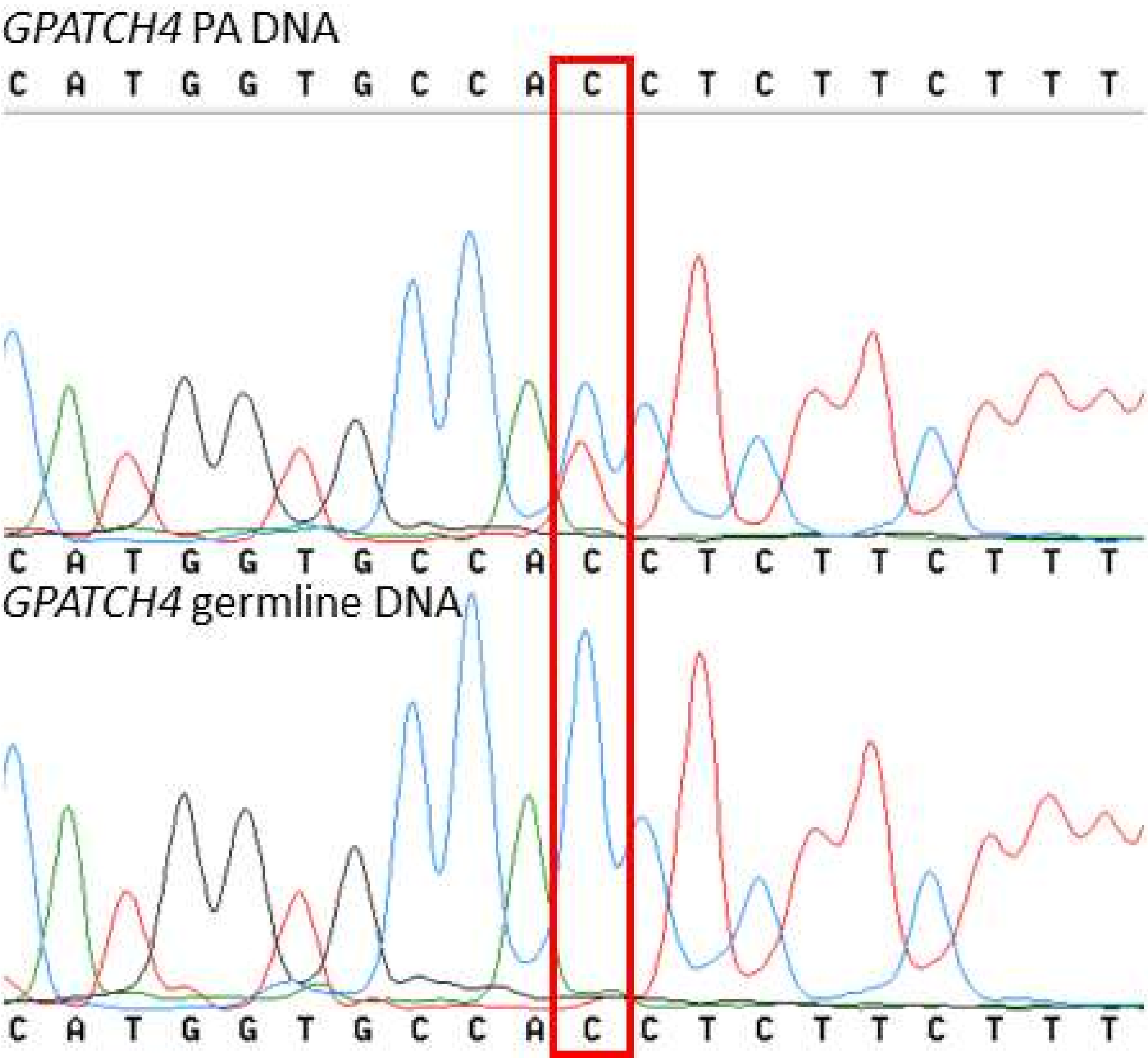

HA066 G6PC2 chr2:169764546

Amplicon sequence:

>chr2:169764509-169764610 102bp

TCCATTCCCCTAACTGTGGTTGctttcattccctactctgttcatatgtt
aatgaaacaaagcggaaagaagagtcagtagAGTGGTGCCTAGAGT
TAGTGC

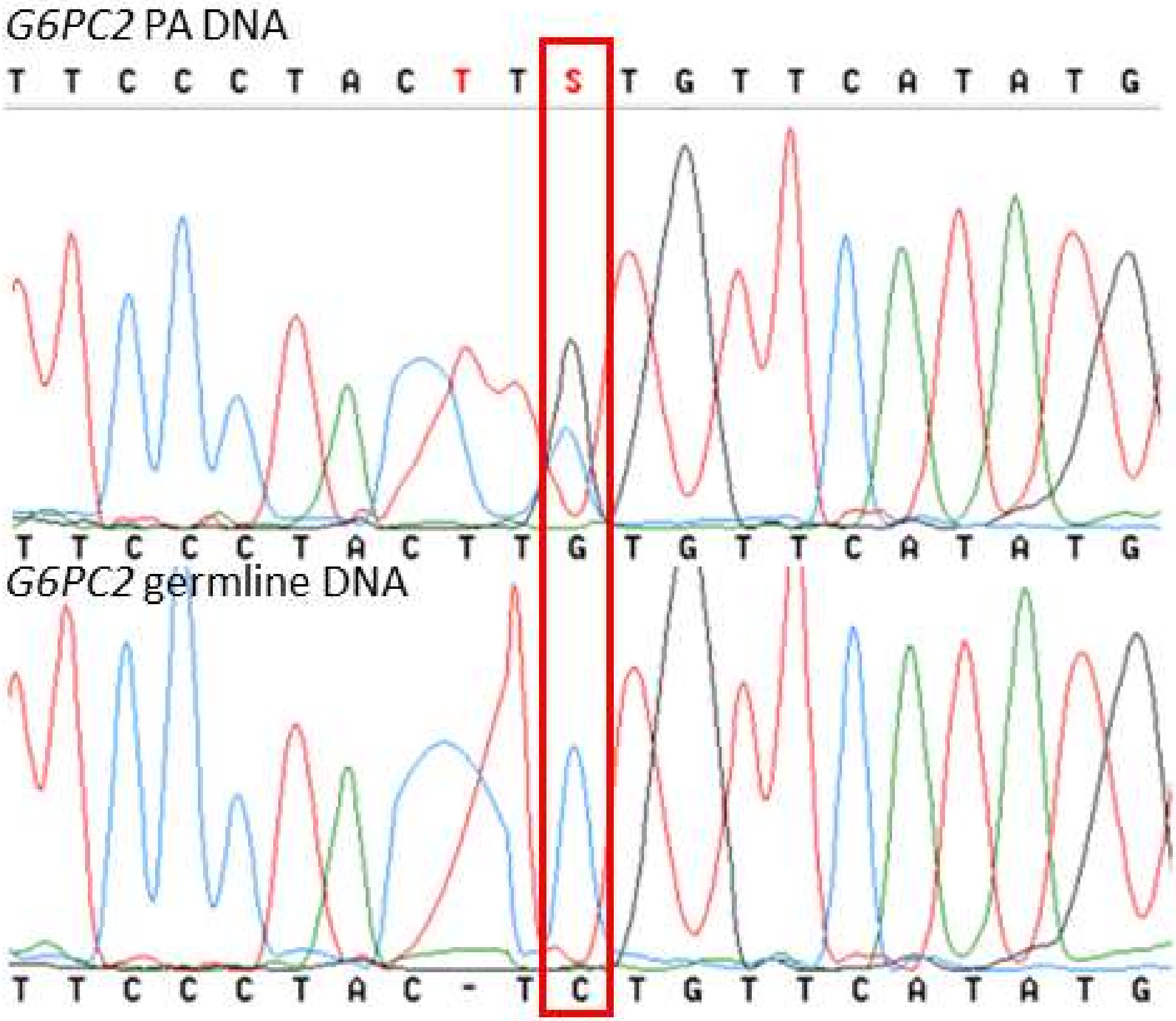

HA073 MPRIP chr17:17068722

Amplicon sequence:

>chr17:17068671-17068776 106bp

CTAGGCCTCCCACACACAAGgctcgaggatgaggacgaggacctg

ggggctcctccgggggaagagtacggtgatggcagccccagTAGGGAA

GACAGCATGGTGC

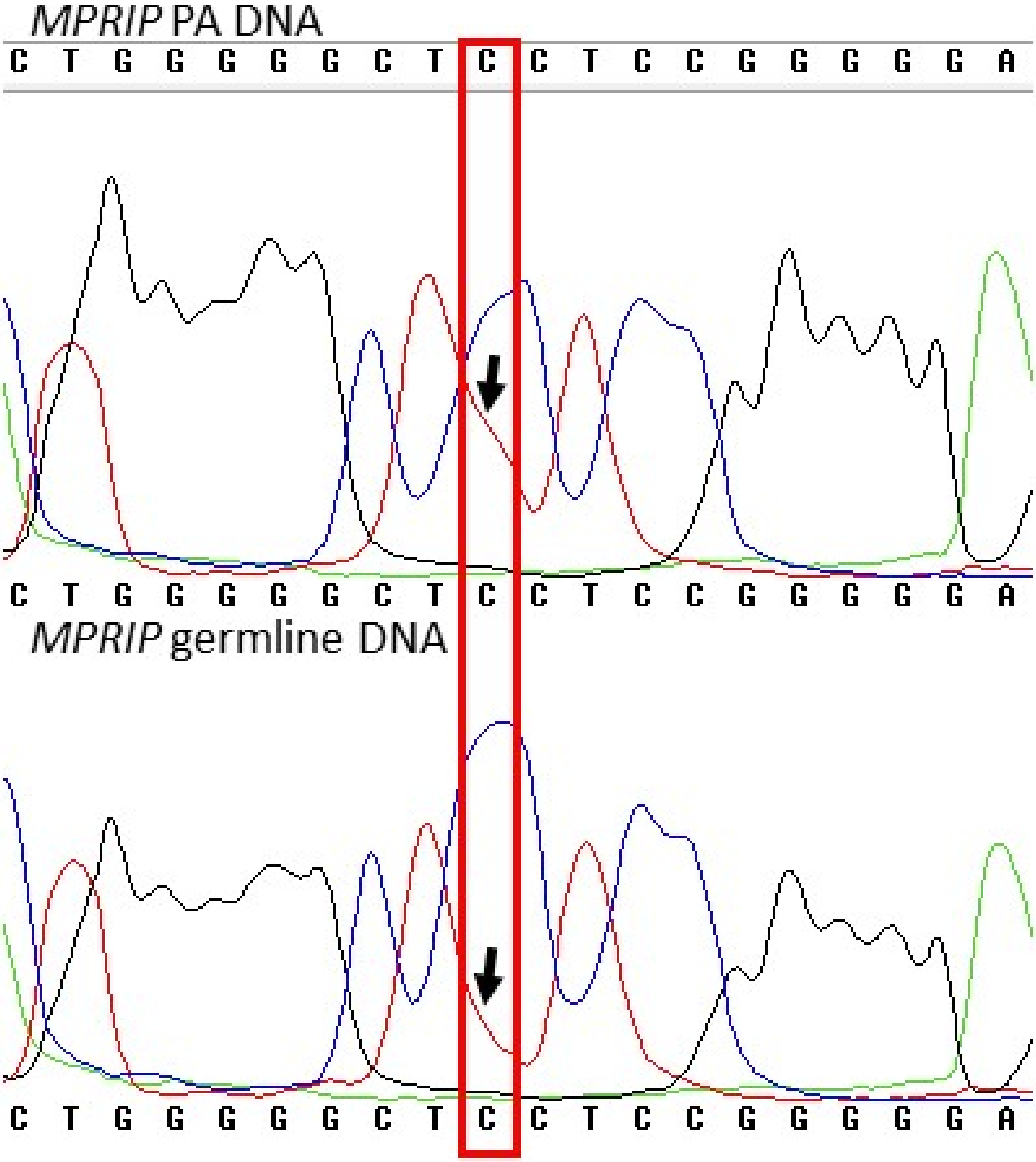

HA070 RYR1 chr19:38964226

Amplicon sequence:

>chr19:38964132-38964256 125bp

TCCAGTTCCACCAGCACTTCcgctgcactgcaggggccaccccgctg
gcacctcctggcctgcagccccccgccgaggacgaggcccgggcggcgga
acc cgaccCTGACTACGAAAACCTGCGC

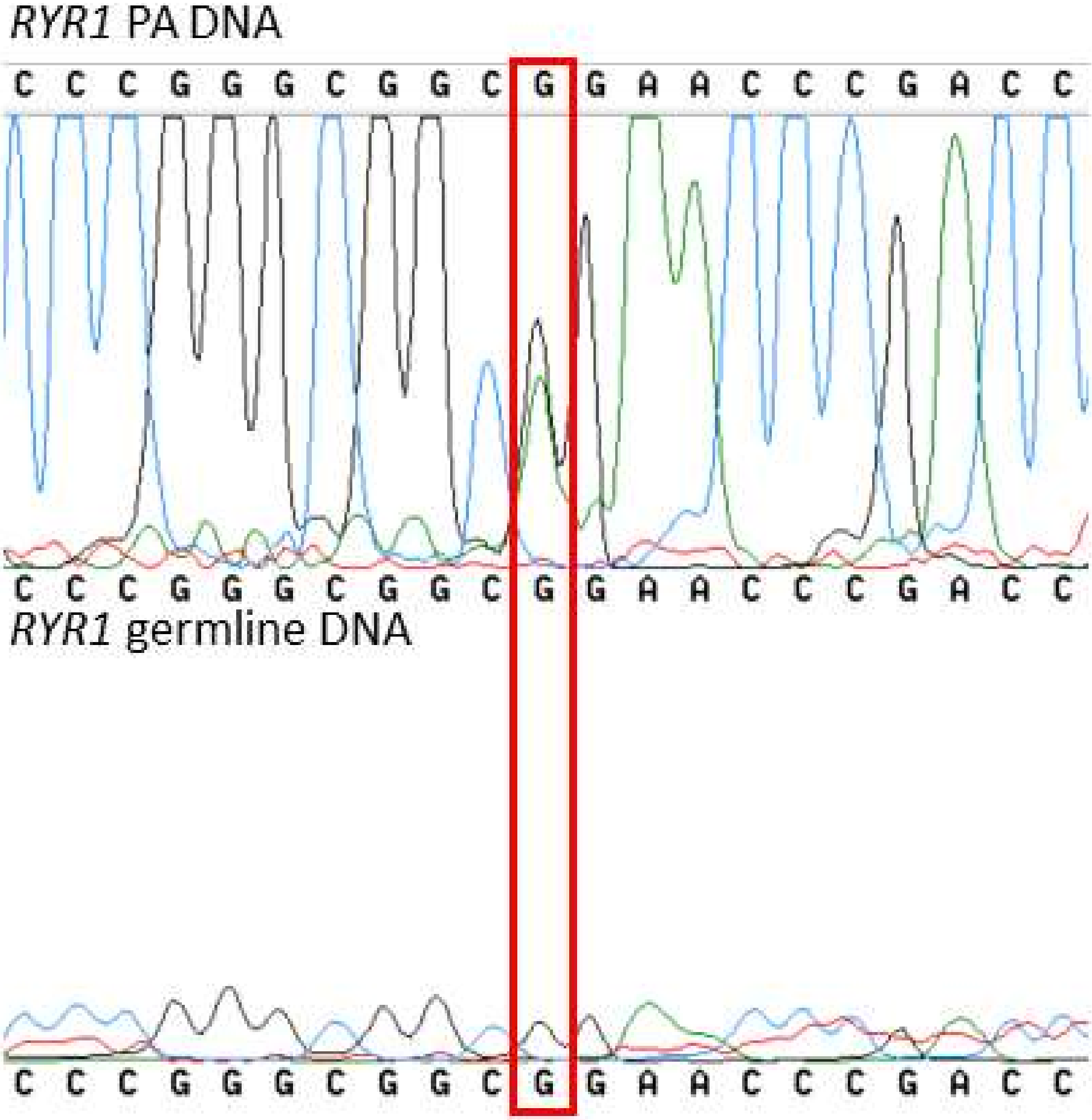

HA090 GNAS chr 20:58909365

Amplicon sequence:

>chr20:57484362-57484466 105bp

CGGTTGGCTTTGGTGAGATCCattgacctcaattttgtttcaggacct
gcttcgctgccgtgtcctgacttctggaatctttgAGACCAAGTTCCAGG
TGGACAA

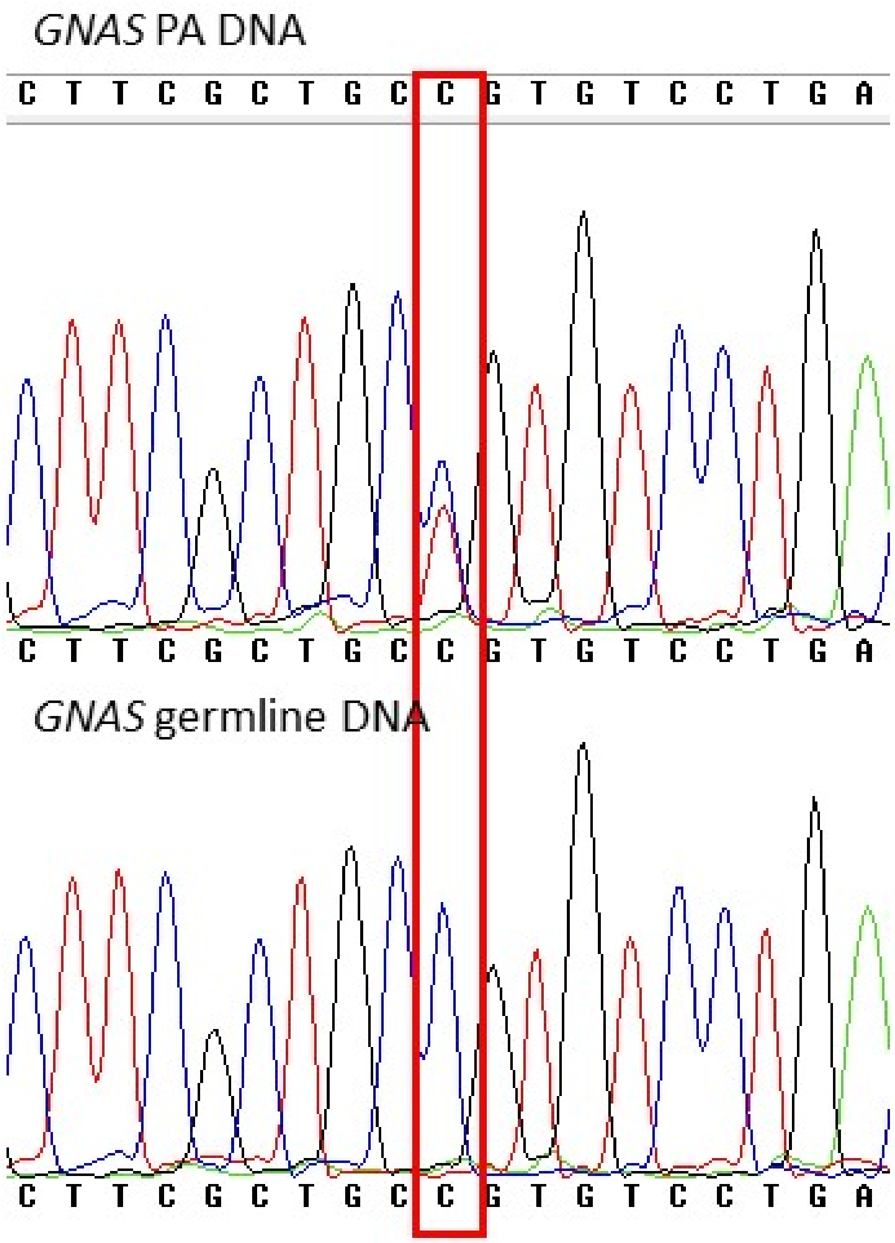

HA070 CLEC1B chr12:10149329

Amplicon sequence:

>chr12:10149275-10149399 125bp

ACCTCAAATGTAACACTTGACCTttttttgttttttacatttttcttaac
atctttcagtttgtactaatctaagacccttgtgaaacctagtaagaattACA
GAACCCAGATATTCAATCCACT

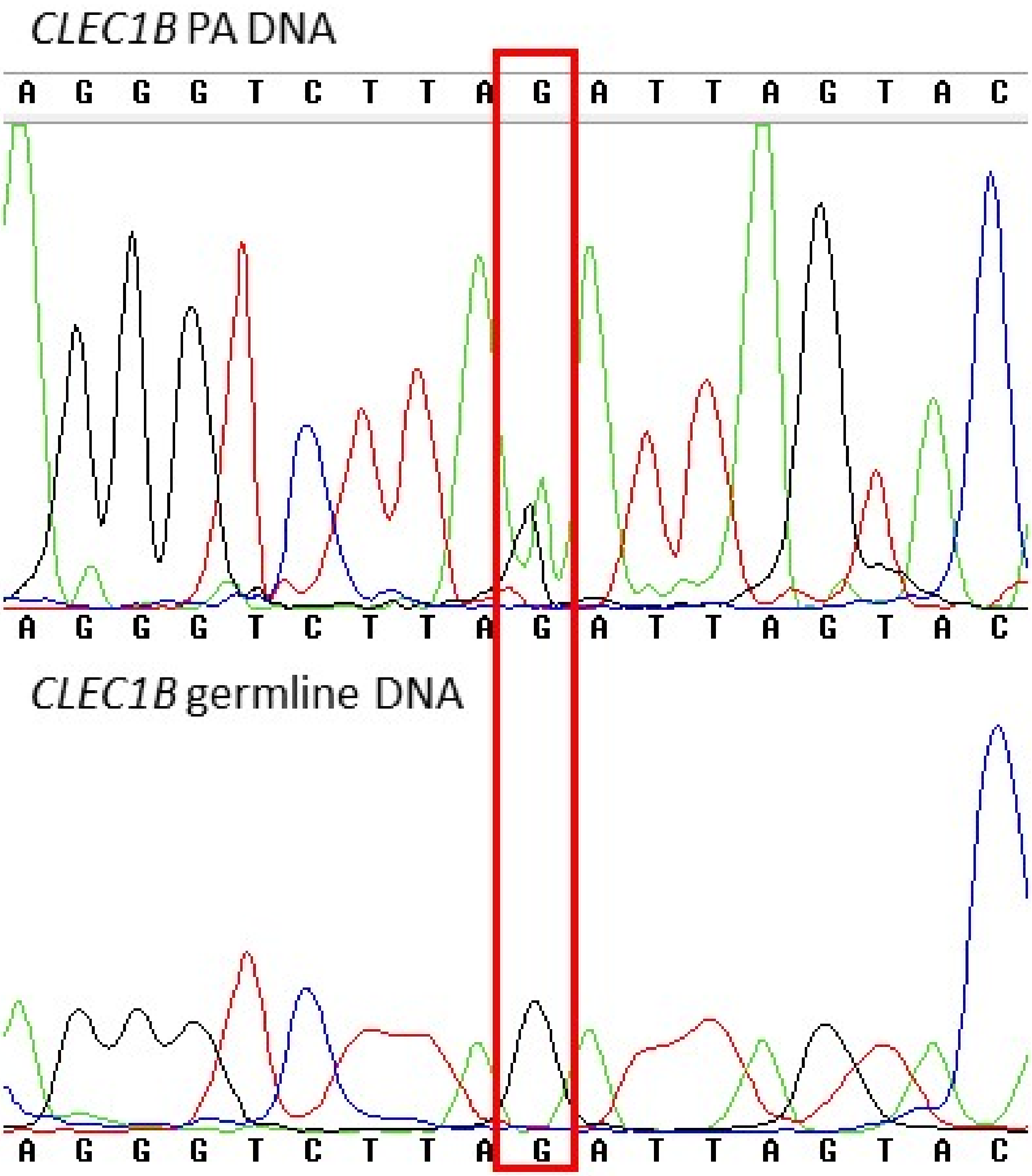

HA073 ATF4 chr22:39918305

Amplicon sequence:

>chr22:39918233-39918337 105bp

TATCTGGGGTCTCCTCAGCAcagcccctctaccaggggctctccaaa
taggagcctcccatctccaggtgttctctgtgggtctgCCCGTCCCAAAC
CTT ACGAT

ATF4 PA DNA

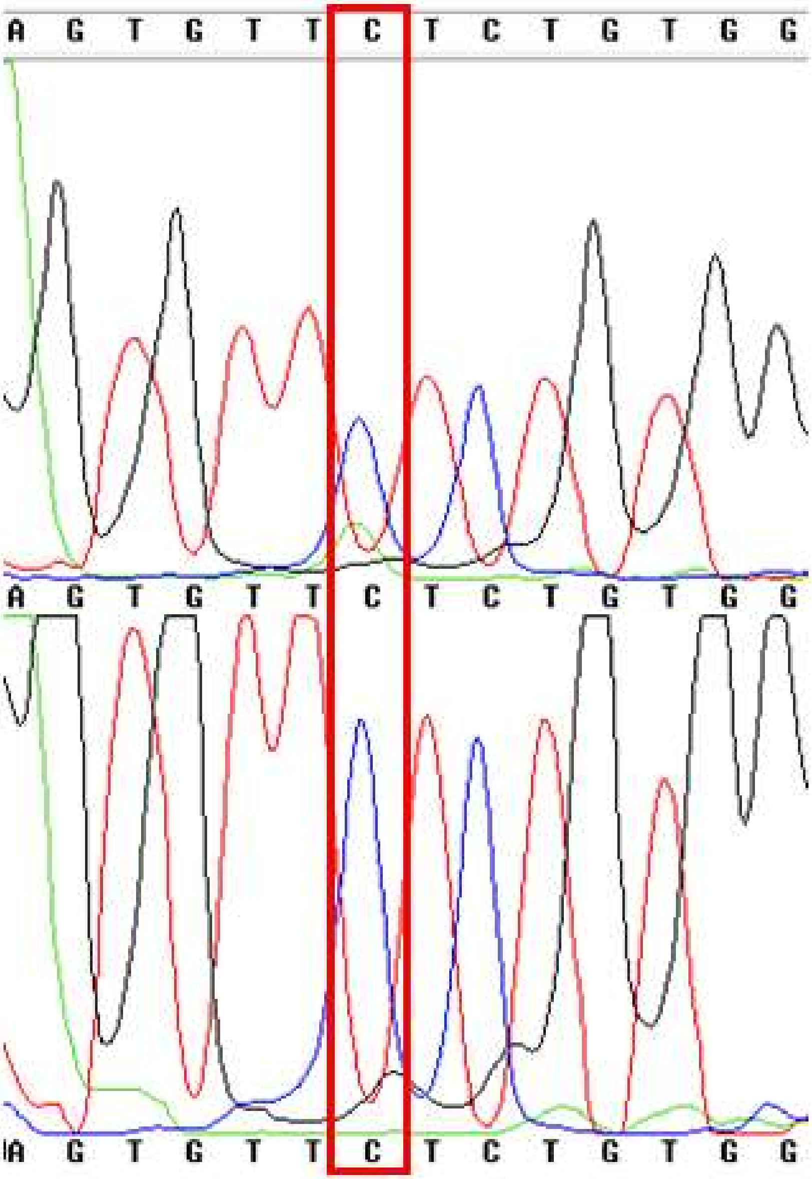

ATF4 germline DNA

HA067 CLCNKA chr1:16358671

Amplicon sequence:

>chr1:16358616-16358730 115bp

CCACCAAGGTCTTCCGGAAGcttggccctcaggcctgtttcttcataa
tg cacctccctccctctccctctctacttgccagagtcccagatcctgGTAG
GCATCGTGCAGAGGG

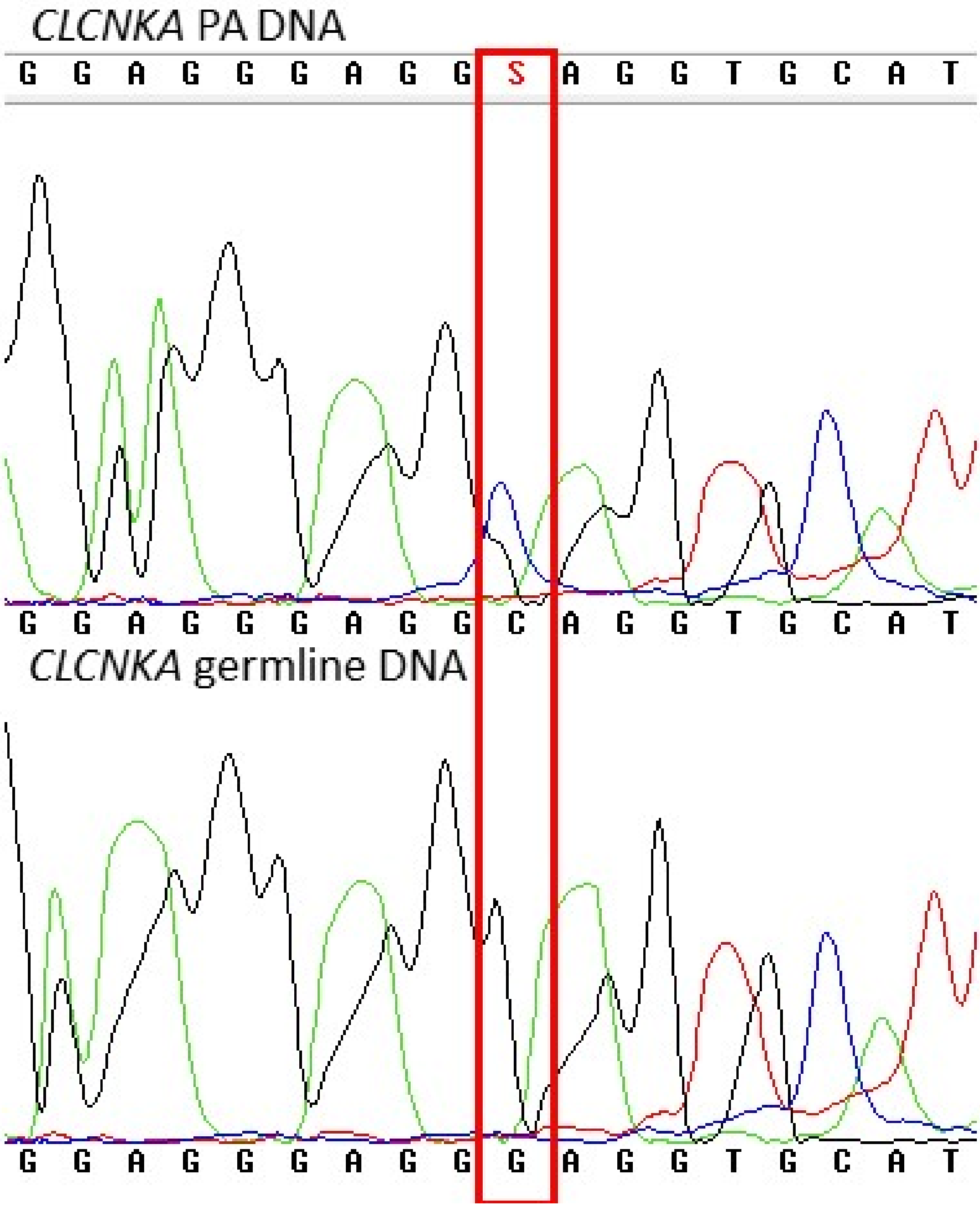

HA067 SMARCAD1 chr4:95174031

Amplicon sequence:

>chr4:95173957-95174075 119bp

TGATTCAGGTTCTGATGTCGGTagttcactagatgaggactatagta
gtggtgaagaagtgatggaggatggctataaaggtaaaattcttcactTCC
TT CAAGATGCTTCAATTGGTG

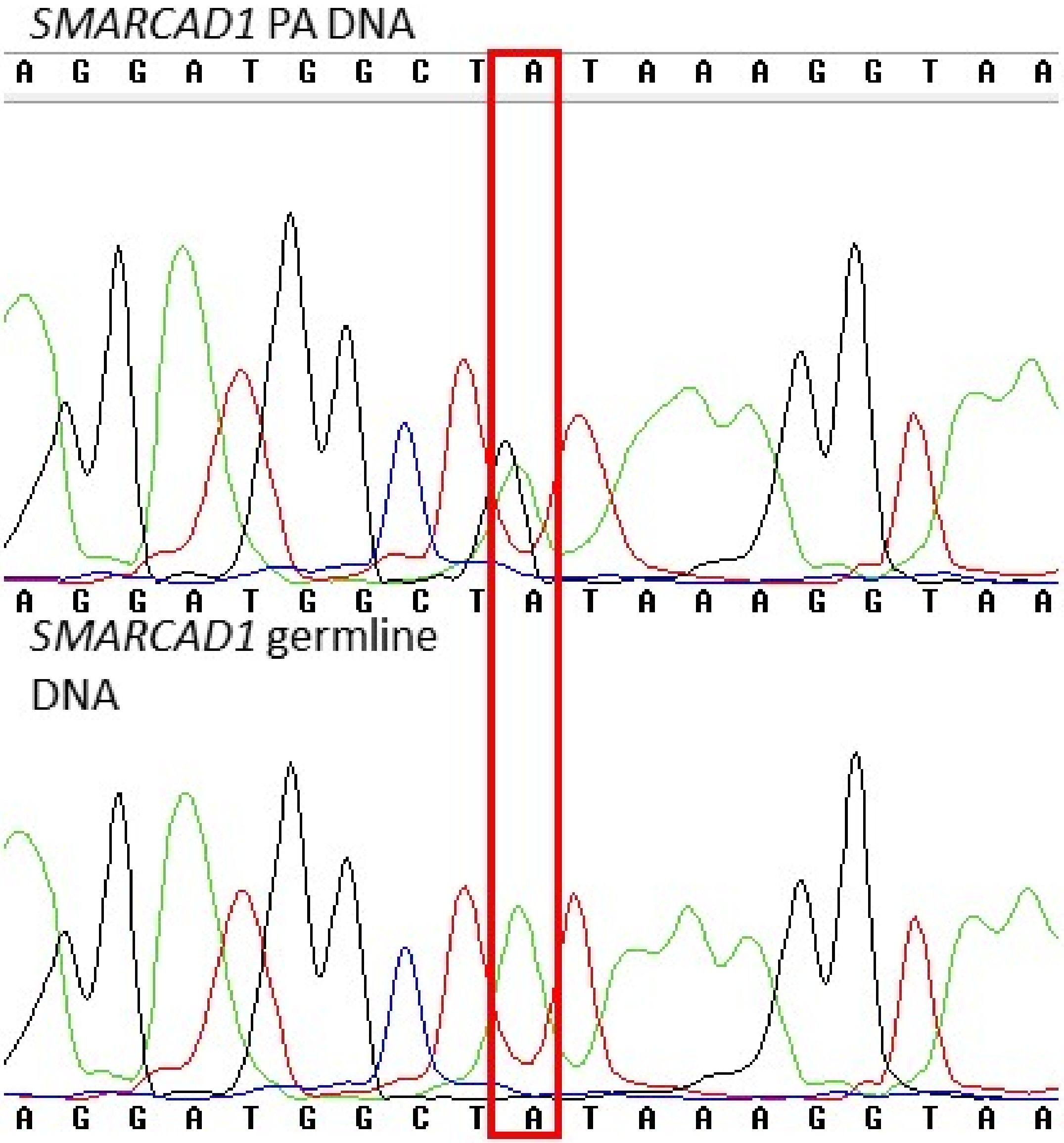

HA067 PDE3A chr12:20774260

Amplicon sequence:

>chr12:20774212-20774347 136bp

TCTTTCTGGTGCTTTTAGTCCTGAttcttggaataatccagtgatgat
gaccctcaccaaaagcagatcctttacttcatcctatgctatttctgcagctaa
ccatgtaaaggctaAAAAGCAAAGTCGACCAGGT

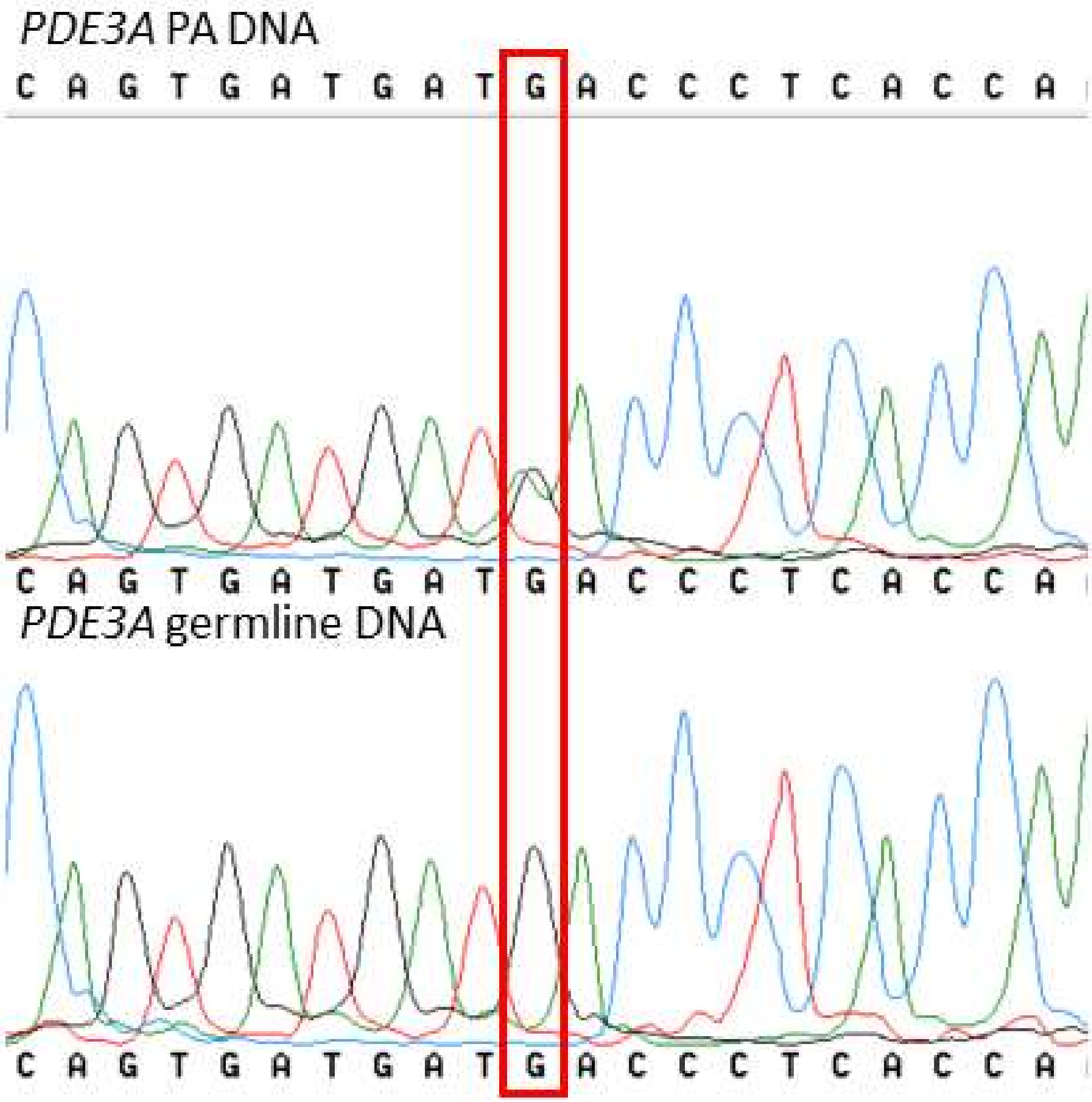

HA067 MTFMT chr15:65316064

Amplicon sequence:

>chr15:65316038-65316138 101bp

ACTCAGTGGCATATTGAATGTTCAtcccagttgcctcccgagatggc
gtggcccagcccctgtaatccatacagtgcttcACGGAGACACAGTTA
CTGGA G

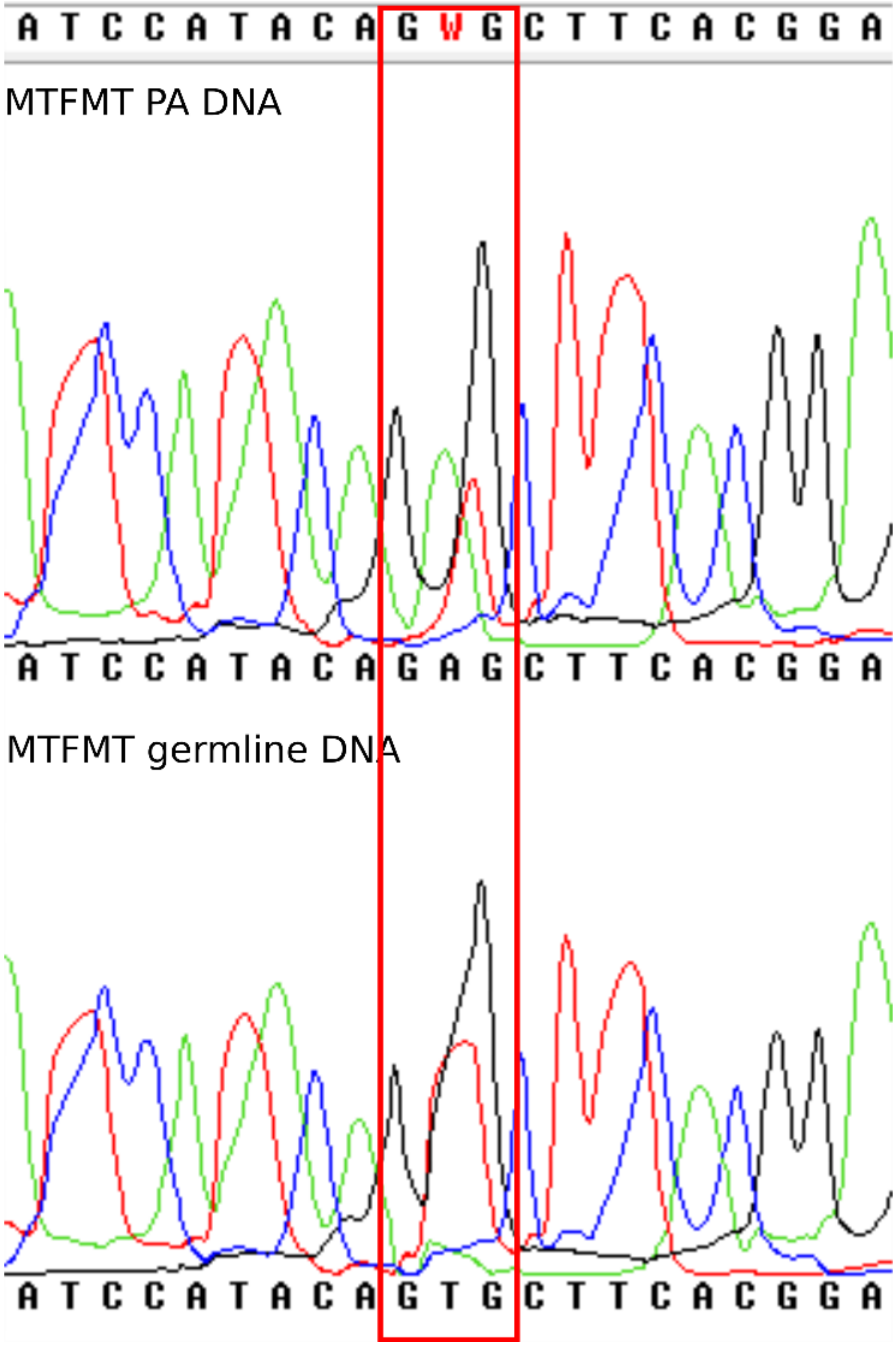

HA070 CCDC138 chr2:109473249

Amplicon sequence:

>chr2:109473212-109473341 130bp

AGCAAAACTGCAGTTGATTGTacataactattttccagctgattacct
ggctcaggcatttgattctctttgtttggacttgaagacagaagaaggaaaaa
ccttgtTTTTGGAGTATCAGGCTGTTCCA

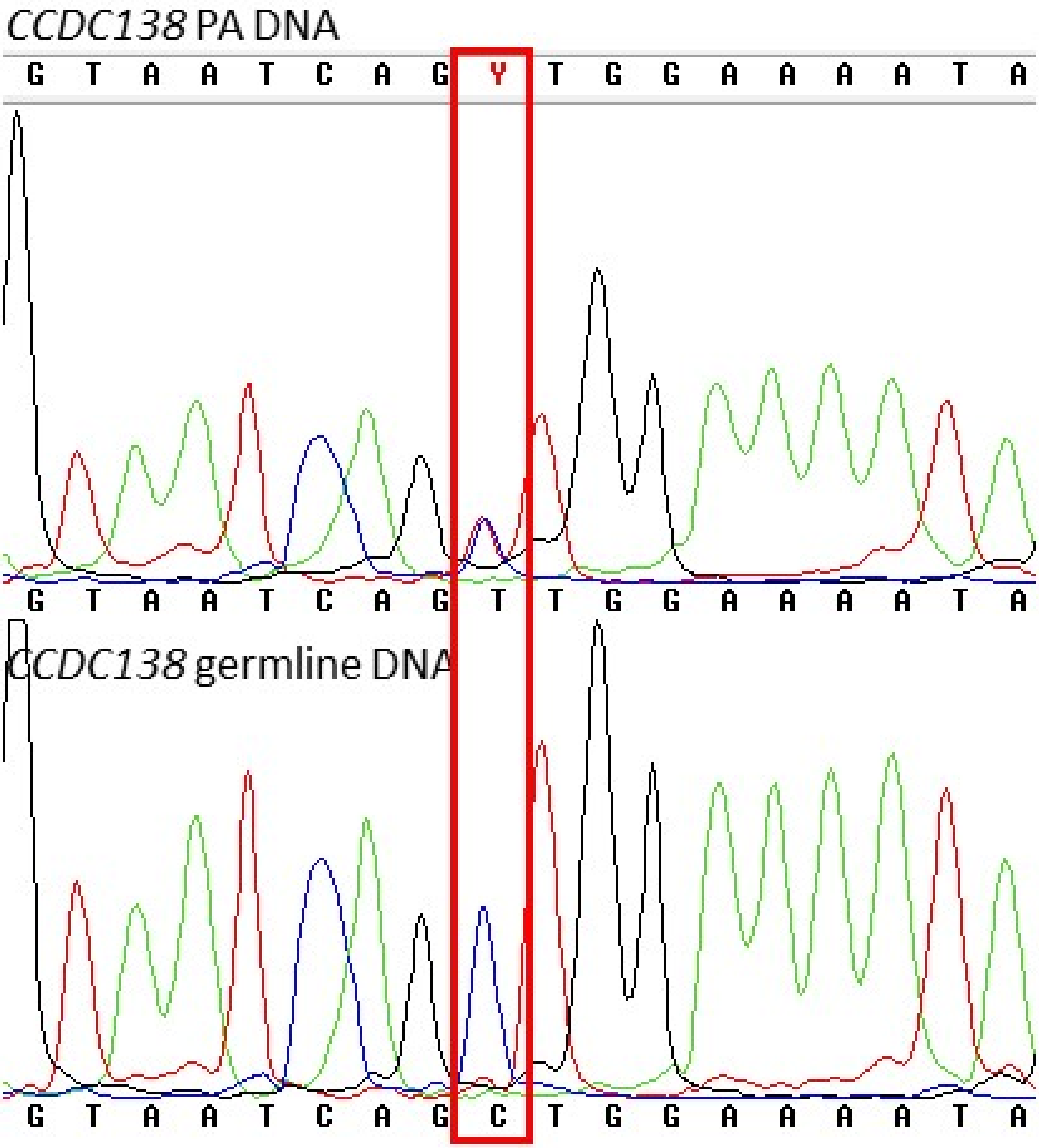

HA070 FXR1 chr3:180633413

Amplicon sequence:

>chr3:180633345-180633461 117bp

AGTTGCCTTGAGATGAGAGCtgttaacattttagtgtatttctttggttt
tttttggtggtttttcaatgtacaggtgatctgaatttcccttttttCCCCTCCT
CTTACCCCGTAT

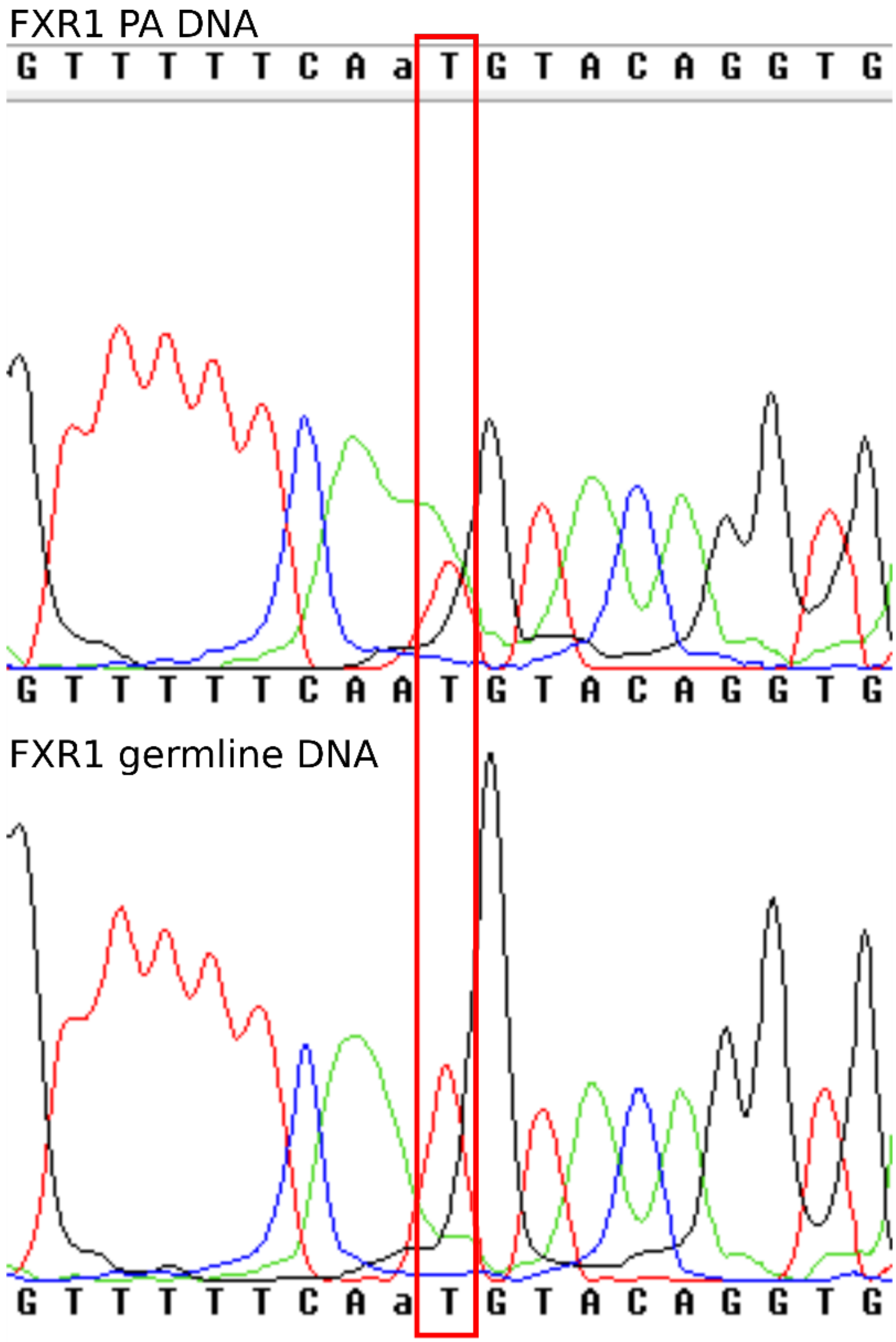

HA067 PRPF8 chr17:1563731

Amplicon sequence:

>chr17:1563708-1563813 106bp

CCCCACGCTGAAGATCTCTCtcatccagatcttccgagctcacttgtg
gcagaagatccatgagagcattgttatggacttatgtcagGTGGGCTGG
AAT CGAGGG

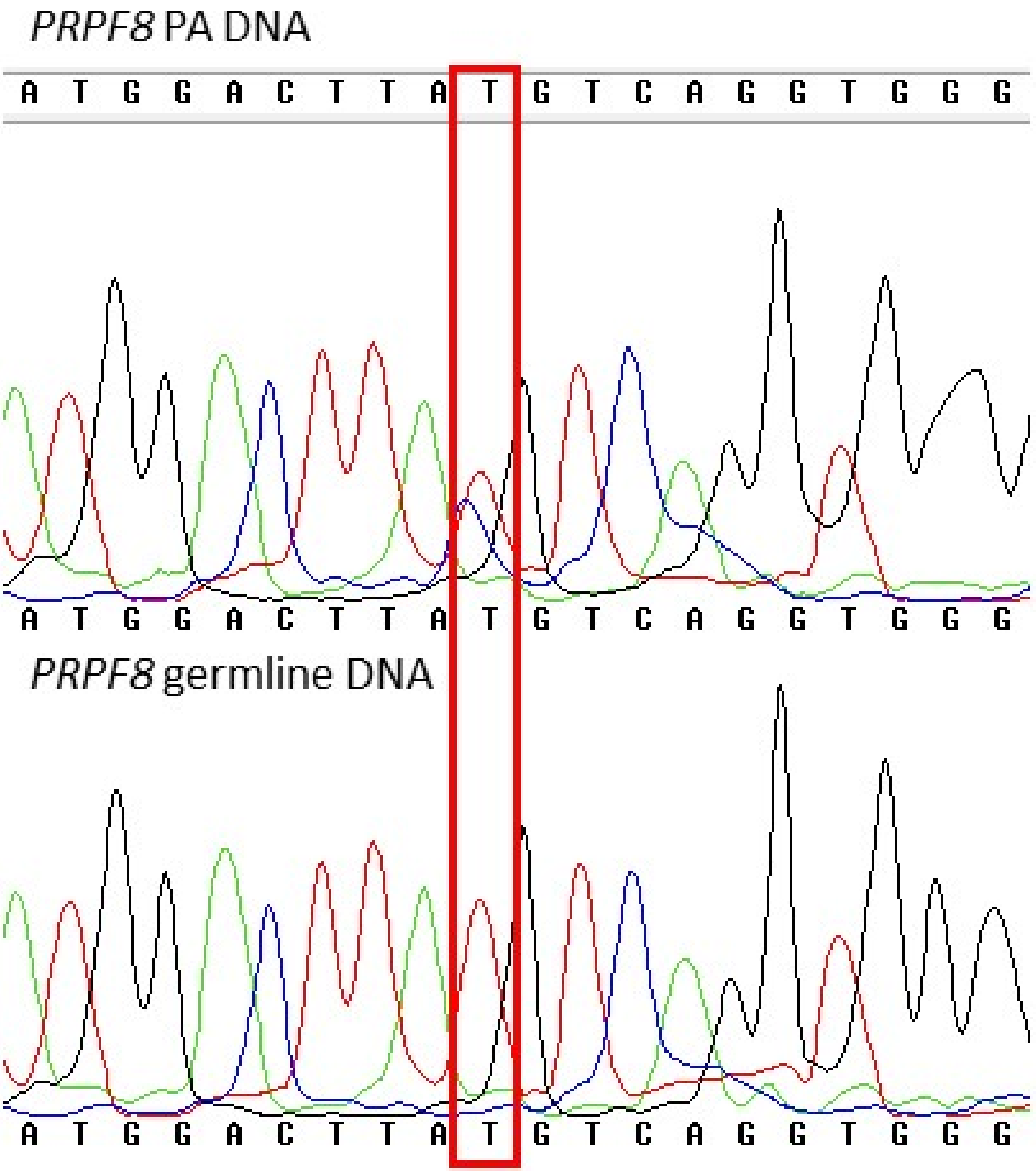

Supplement: Supplementary Figure 2 — Validation of LDLRAD2, SPEN, VPS13D, G6PC2, GPATCH4, CLCNKA, MTFMT, PDE3A, PRPF8, SMARCAD1, CCDC138, CLEC1B, FXR1, RYR1, ATF4, MPRIP gene variants with Sanger sequencing in pituitary adenoma somatic (PA) DNA and patients germline DNA from samples HA065, HA066, HA067, HA070, HA073, and HA090. [file Data_Sheet_1.PDF]
