# Supplementary material for: Evaluation of the Possibility to Detect Circulating Tumor DNA From Pituitary Adenoma
Source: Front Endocrinol (Lausanne). 2019 Sep 18;10:615. doi: 10.3389/fendo.2019.00615 (PMC6759656; doi:10.3389/fendo.2019.00615)
Supplement: Supplementary Table 1 — Used primers (Metabion international AG, Germany). [file Table_1.DOCX]

**Supplementary Table 1.** Used primers (Metabion international AG, Germany)

| Name | Nucleotide sequence (5’ - 3’) |
| --- | --- |
| GNAS_Fw | CGGTTGGCTTTGGTGAGATCC |
| GNAS_Rs | TTGTCCACCTGGAACTTGGTCT |
| GNAS227_Fw | CTGTCCTCCTCCCCACCA |
| GNAS227_Rs | AACAGCCAAGCCCACAGC |
| SMARCAD1_Fw | TGATTCAGGTTCTGATGTCGGT |
| SMARCAD1_Rs | AGGAAGTTCTACGAAGTTAACCAC |
| PDE3A_Fw | TCTTTCTGGTGCTTTTAGTCCTGA |
| PDE3A_Rs | TTTTCGTTTCAGCTGGTCCA |
| MTFMT_Fw | ACTCAGTGGCATATTGAATGTTCA |
| MTFMT_Rs | TGCCTCTGTGTCAATGACCTC |
| RYR1_Fw | TCCAGTTCCACCAGCACTTC |
| RYR1_Rs | GACTGATGCTTTTGGACGCG |
| CCDC138_Fw | AGCAAAACTGCAGTTGATTGT |
| CCDC138_Rs | AAAACCTCATAGTCCGACAAGGT |
| FXR1_Rs | AGTTGCCTTGAGATGAGAGC |
| FXR1_Rs | GGGGAGGAGAATGGGGCATA |
| PRPF8_Fw | CCCCACGCTGAAGATCTCTC |
| PRPF8_Rs | CACCCGACCTTAGCTCCC |
| VPS13D_Fw | TGGATTGCGTTGTCGTGGAT |
| VPS13D_Rs | AAACACGCTGTCTGTCCTCC |
| LDLRAD2_Fw | CCCCTGGCTTCAAGTTCTGT |
| LDLRAD2_Rs | GGAATGGCGTCACGACAGT |
| SPEN_Fw | TCGTTGGGACTCTCAGATGA |
| SPEN_Rs | TTCCAGACATGCTCTAGACCT |
| GPATCH4_Fw | GGGAATGAGAAGGAGGACGC |
| GPATCH4_Rs | TTCTACCCCCAGAACCTCCT |
| G6PC2_Fw | TCCATTCCCCTAACTGTGGTTG |
| G6PC2_Rs | TCACCACGGATCTCAATCACG |
| MPRIP_Fw | CTAGGCCTCCCACACACAAG |
| MPRIP_Rs | ATCCCTTCTGTCGTACCACG |
| CLEC1B_Fw | ACCTCAAATGTAACACTTGACCT |
| CLEC1B_Rs | TGTCTTGGGTCTATAAGTTAGGTGA |
| ATF4_Fw | TATCTGGGGTCTCCTCAGCA |
| ATF4_Rs | GGGCAGGGTTTGGAATGCTA |
| CLCNKA_Fw | CCACCAAGGTCTTCCGGAAG |
| CLCNKA_Rs | CATCCGTAGCACGTCTCCC |
